# Serum MicroRNAs Predict Isolated Rapid Eye Movement Sleep Behavior Disorder and Lewy Body Diseases

**DOI:** 10.1002/mds.29171

**Published:** 2022-08-12

**Authors:** Marta Soto, Alex Iranzo, Sara Lahoz, Manel Fernández, Mónica Serradell, Carles Gaig, Paula Melón, Maria‐Jose Martí, Joan Santamaría, Jordi Camps, Rubén Fernández‐Santiago, Mario Ezquerra

**Affiliations:** ^1^ Laboratory of Parkinson Disease and Other Neurodegenerative Movement Disorders, Institut d'Investigacions Biomèdiques August Pi i Sunyer (IDIBAPS)‐Hospital Clínic de Barcelona University of Barcelona Barcelona Spain; ^2^ Centro de Investigación Biomédica en Red de Enfermedades Neurodegenerativas (CIBERNED) Barcelona Spain; ^3^ Sleep Center, Department of Neurology, Hospital Clínic de Barcelona, Institut d'Investigacions Biomèdiques August Pi i Sunyer (IDIBAPS) University of Barcelona Barcelona Spain; ^4^ Gastrointestinal and Pancreatic Oncology Team, Institut d'Investigacions Biomèdiques August Pi i Sunyer (IDIBAPS)‐Hospital Clínic de Barcelona Barcelona Spain; ^5^ Centro de Investigación Biomédica en Red de Enfermedades Hepáticas y Digestivas (CIBERehd) Madrid Spain; ^6^ Movement Disorders Unit, Department of Neurology, Hospital Clínic de Barcelona, Institut d'Investigacions Biomèdiques August Pi i Sunyer (IDIBAPS) University of Barcelona Barcelona Spain

**Keywords:** isolated REM‐sleep behavior disorder (IRBD), dopamine transporter single‐photon emission computed tomography (DaT‐SPECT), biomarkers, disease prediction, Parkinson's disease (PD)

## Abstract

**Background:**

Isolated rapid eye movement sleep behavior disorder (IRBD) is a well‐established clinical risk factor for Lewy body diseases (LBDs), such as Parkinson's disease (PD) and dementia with Lewy bodies (DLB).

**Objective:**

To elucidate whether serum microRNA (miRNA) deregulation in IRBD can antedate the diagnosis of LBD by performing a longitudinal study in different progression stages of IRBD before and after LBD diagnosis and assessing the predictive performance of differentially expressed miRNAs by machine learning–based modeling.

**Methods:**

Using genome‐wide miRNA analysis and real‐time quantitative polymerase chain reaction validation, we assessed serum miRNA profiles from patients with IRBD stratified by dopamine transporter (DaT) single‐photon emission computed tomography into DaT‐negative IRBD (n = 17) and DaT‐positive IRBD (n = 21), IRBD phenoconverted into LBD (n = 13), and controls (n = 20). Longitudinally, we followed up the IRBD cohort by studying three time point serum samples over 26 months.

**Results:**

We found sustained cross‐sectional and longitudinal deregulation of 12 miRNAs across the RBD continuum, including DaT‐negative IRBD, DaT‐positive IRBD, and LBD phenoconverted IRBD (let‐7c‐5p, miR‐19b‐3p, miR‐140, miR‐22‐3p, miR‐221‐3p, miR‐24‐3p, miR‐25‐3p, miR‐29c‐3p, miR‐361‐5p, miR‐425‐5p, miR‐4505, and miR‐451a) (false discovery rate *P* < 0.05). Age‐ and sex‐adjusted predictive modeling based on the 12 differentially expressed miRNA biosignatures discriminated IRBD and PD or DLB from controls with an area under the curve of 98% (95% confidence interval: 89–99%).

**Conclusions:**

Besides clinical diagnosis of IRBD or imaging markers such as DaT single‐photon emission computed tomography, specific miRNA biosignatures alone hold promise as progression biomarkers for patients with IRBD for predicting PD and DLB clinical outcomes. Further miRNA studies in other PD at‐risk populations, such as LRRK2 mutation asymptomatic carriers or hyposmic subjects, are warranted. © 2022 The Authors. *Movement Disorders* published by Wiley Periodicals LLC on behalf of International Parkinson and Movement Disorder Society

Rapid eye movement (REM) sleep behavior disorder (RBD) is characterized by the lack of REM sleep–related muscle atonia, disinhibited motor activity, and dream‐enacting behaviors. The isolated form of RBD (IRBD) represents the prodromal state of the synucleinopathies because, after 14 years of follow‐up, 91% of the patients with IRBD are diagnosed with Parkinson's disease (PD) and dementia with Lewy bodies (DLB), collectively known as Lewy body disorders (LBDs), and rarely with multiple system atrophy (MSA).^1^ Yet, predicting disease progression in IRBD is challenging given that time to phenoconversion is variable and the resulting clinicopathological phenotype is heterogeneous, being DLB and MSA rapidly progressive diseases compared with PD. Thus, investigating IRBD can help assess the earliest molecular changes occurring at prodromal stages of α‐synucleinopathies and identify potential diagnostic or prognostic biomarkers for these diseases.

MicroRNAs (miRNAs) are small noncoding RNAs that regulate gene expression by mRNA cleavage and translation repression.^2^ Cumulative evidence showed serum or plasma miRNA deregulation in patients with PD and related α‐synucleinopathies,^3^, ^4^, ^5^, ^6^, ^7^, ^8^ including its prodromal phases such as IRBD.^9^, ^10^ Yet, miRNA studies in premotor cohorts are still scarce, and longitudinal follow‐ups in seriated IRBD progression stages are needed. In this study, we characterized the serum miRNA expression profiles of a Spanish IRBD cohort without motor and cognitive impairment and segregated by dopamine transporter single‐photon emission computed tomography (DaT‐SPECT) into DaT‐negative IRBD, DaT‐positive IRBD, IRBD phenoconverted into PD and DLB, and controls. We used this method to stratify IRBD into successive disease progression stages, given that abnormal DaT‐SPECT represents a short‐term marker for developing PD and DLB in less than 5 years.^11^, ^12^, ^13^ Cross‐sectionally, we identified novel differentially expressed miRNAs (DEmiRs) associated with IRBD and α‐synucleinopathy, which we subsequently validated using three longitudinal follow‐up serum samples from DaT‐negative and DaT‐positive IRBD. Lastly, by applying machine learning modeling algorithms, we assessed the disease prediction ability of the identified DEmiR to discriminate IRBD and PD or LBD patients from controls alone. The goal of the study was to investigate potential miRNAs as prognostic biomarkers for α‐synucleinopathies.

## Subjects and Methods

### Subjects

All subjects provided written informed consent, and the Ethics Committee of Institut d'Investigacions Biomèdiques August Pi i Sunyer (IDIBAPS)‐Hospital Clínic de Barcelona approved the study. The IRBD diagnosis required a history of dream‐enacting behaviors, audiovisual‐polysomnographic confirmation of excessive electromyographic activity in REM sleep, and absence of motor and cognitive impairment.^1^ All the participating IRBD subjects were characterized by dopamine transporter single‐photon emission computed tomography (DaT‐SPECT) imaging using ^123^I‐2β‐carbomethoxy‐3β‐(4‐iodophenyl)‐N‐(3‐fluoropropyl)‐nortropane as previously described.^13^ Overall, the cross‐sectional cohort consisted of 51 patients with IRBD recruited and diagnosed at the Sleep Centre of the Neurology Service at the Hospital Clínic de Barcelona, Spain. Of these, 38 patients with IRBD were free of neurodegenerative disease, 17 of which had normal DaT‐SPECT (DaT‐negative IRBD) and 21 abnormal DaT‐SPECT (DaT‐positive IRBD). The remaining 13 patients were initially diagnosed with IRBD, and clinical follow‐up showed the phenoconversion into LBD, PD (n = 8), and DLB (n = 5), fulfilling accepted criteria.^14^, ^15^ The study included 20 healthy control patients without evidence of neurological or sleep disorders from the Parkinson's Disease and Movement Disorders Unit at the Hospital Clínic de Barcelona (Table 1). Basal RBD and LBD medication is summarized in Table S1. All patients with IRBD were clinically followed up every 3–12 months, and serum samples were collected for at least three time points over 26 months (Table S2). During this period, three DaT‐positive IRBD subjects (14%) were diagnosed with PD, one between the first and the second time point and two between the second and the third.

**TABLE 1:** Clinicodemographics of the study subjects

Group	No. of subjects	Sex (M/F)	Age at IRBD onset (y)	Age at sampling (y)	Time from IRBD onset to sampling (y)	Interval from baseline (mo)	No. converted to PD	Age at PD diagnosis	No. converted to DLB	Age at DLB diagnosis (y)
Controls	20	16/4	–	70.10 ± 6.19	–	–	–	–	–	–
DaT(−) IRBD	17	14/3	63.65 ± 8.73	71.12 ± 8.21	7.12 ± 5.57	–	–	–	–	–
Time point 2	16	11/2	64.06 ± 8.84	72.50 ± 8.14	8.38 ± 5.80	10.13 ± 2.68	–	–	–	–
Time point 3	14	10/1	65.29 ± 8.79	74.50 ± 7.81	9.00 ± 5.66	19.57 ± 4.82	–	–	–	–
DaT(+) IRBD	21	16/4	61.38 ± 7.98	70.57 ± 5.73	8.86 ± 7.58	–	–	–	–	–
Time point 2	19	13/4	61.84 ± 8.26	71.95 ± 5.79	9.58 ± 7.71	10.84 ± 2.14	1	76	–	–
Time point 3	19	14/4	61.84 ± 8.26	72.39 ± 6.12	10.32 ± 7.72	20.00 ± 4.00	3	72.67 ± 4.93	–	–
LBD	13	7/6	60.15 ± 9.26	76.77 ± 5.34	16.62 ± 8.30	–	8	73.88 ± 5.77	5	76.40 ± 4.83

Clinicodemographic data from DaT‐negative IRBD, DaT‐positive IRBD, IRBD phenoconverted into LBD (PD and DLB), and healthy controls.

M, male; F, female; IRBD, isolated rapid eye movement sleep behavior disorder; PD, Parkinson's disease; DLB, dementia with Lewy bodies; LBD, Lewy body disease (PD and DLB); DaT, dopamine transporter‐single‐photon emission computed tomography imaging; DaT(−) IRBD, dopamine transporter‐negative IRBD patients; DaT(+) IRBD, dopamine transporter‐positive IRBD patients.

### Serum miRNA Isolation

For each subject, we collected 5 mL peripheral blood in tubes with a clot activator (#366468; BD Vacutainer), preserved it for 30 minutes at room temperature, and centrifuged at 1500*g* for 10 minutes at 4°C. Serum volumes of 2 mL were removed from the supernatant, aliquoted in polypropylene CryoTubes (#121263; Greiner Bio‐One), and stored at −80°C. We mixed 200 μL serum from each sample with 2 μL of yeast transfer RNA (tRNA) (#AM7119; Invitrogen) at a final concentration of 10 μg/mL as a carrier for nucleic acid precipitation. Following the manufacturer's instructions, total RNA enriched in miRNAs was isolated using the miRNeasy Serum/Plasma Kit (#217184; QIAGEN). The RNA concentration was determined on a NanoDrop ND‐3300 fluorospectrometer (Thermo Scientific). We performed an electropherogram analysis using an Agilent Small RNA Kit in n = 22 random samples as quality control for 6‐ to 40‐bp miRNA‐enriched fractions and observed high‐quality miRNA enrichment for all studied samples (Fig. S1).

### Genome‐Wide miRNA Profiling

We explored the genome‐wide miRNA expression profiles in serum using the Affymetrix GeneChip miRNA 4.0 Array, which interrogates the levels of 4603 human miRNAs, including 2578 mature miRNAs and 2025 pre‐miRNAs (#902413; Thermo Fisher) (Product datasheet: https://www.thermofisher.com/). Serum miRNAs from individual subjects were hybridized onto the probe set separate arrays for 42 hours and scanned using an Affymetrix GeneChip Scanner 3000 7G following the manufacturer's instructions. Data files generated by the Affymetrix GeneChip Command Console were processed with the Expression Console software to determine the data quality. Expression raw data were analyzed using the Partek Genomic Suite v7.0 software (Partek Inc.) applying the robust multiarray average background correction model, which permits the relative comparison of miRNA abundance in different arrays. Only miRNAs with detection values greater than 2.4 arbitrary luminescence units in at least 50% of all samples, patients, and controls and a significant expression value above the background signal (*P* < 0.05) were considered as expressed in serum. In line with previous reports,^16^ we observed that approximately 10% of the mature human screened miRNAs were expressed in serum from our cohort. Adjusting by sex and age, we used the global mean of all expressed serum miRNAs as a reference to define candidate differentially expressed miRNAs (DEmiRs) and the sign of the fold‐change difference. Under a 2‐tailed Student *t* test, we applied the criteria of a combination of a fold‐change > |1.5| and *P* < 0.05 to select candidate miRNAs from the array for subsequent real‐time quantitative polymerase chain reaction (RT‐qPCR) validation (Table S3).

### RT‐qPCR Validation

miRNA samples were reverse transcribed into cDNA and preamplified using the TaqMan Advanced miRNA cDNA Synthesis Kit (#A28007; Thermo Fisher Scientific) in a Veriti 96‐well Thermal Cycle (Applied Biosystems). cDNA preamplified products were quantified per duplicate using TaqMan Fast Advanced Master Mix (4444557; Thermo Fisher Scientific) and TaqMan Advanced miRNA Assays (A25576; Thermo Fisher Scientific) (Table S4). Reactions were plated with evenly balanced group samples in 96‐well RT‐qPCR plates at a final volume of 10 μL on a TaqMan StepOnePlus RT‐qPCR System (Applied Biosystems). We used two endogenous and one exogenous miRNA simultaneously for normalization in all RT‐qPCR comparisons.^17^, ^18^ As endogenous normalizers, we selected hsa‐miR‐320a‐3p and hsa‐miR‐6727‐5p among the most stable miRNAs from the array across all samples as identified using the NormFinder software^19^ in other α‐synucleinopathies studies in our population, i.e., MSA^20^ and LRRK2‐associated PD (Marta Soto, Manel Fernández, Paloma Bravo, Alicia Garrido, Antonio Sánchez‐Rodríguez, María Rivera‐Sánchez, María Sierra, Paula Melón, Anna Naito, Bradford Casey, Eduardo Tolosa, María‐José Martí, Jon Infante, Mario Ezquerra and Rubén Fernández‐Santiago). As an exogenous normalizer, we used the *Caenorhabditis elegans* miRNA cel‐miR‐39‐3p added to all serum samples at a final concentration of 5 pM.^18^ To assess miRNA relative expression, we used the DataAssist v3.0 software (Applied Biosystems), setting the maximum allowable cycle threshold value at 35 cycles. Statistical significance levels for DEmiRs were set at a fold‐change > |1.5| and a false discovery rate (FDR) adjusted *P* < 0.05 under a 2‐tailed Student *t* test. Using commercially available assays, we tested 22 miRNAs and discarded 7, which did not show an amplification signal. Of the remaining 15 miRNAs (Table S4), 10 were selected among the top candidate miRNAs found by array (let‐7c‐5p, miR‐1207‐5p, miR‐1227‐5p, miR‐140‐3p, miR‐24‐3p, miR‐25‐3p, miR‐361‐5p, miR‐3613‐5p, miR‐425‐5p, and miR‐451a) (Table S5). The remaining five miRNAs were candidates earlier reported in PD alone (miR‐22‐3p, miR‐29c‐3p, miR‐221‐3p, and miR‐4505)^7^, ^21^, ^22^ or both PD and IRBD (miR‐19b‐3p).^8^, ^9^ Lastly, as quality control, we repeated the entire RT‐qPCR experiment in all samples for three of the identified DEmiRs (miR‐140‐3p, miR‐19b‐3p, and miR‐29c‐3p) to discard potentially confounding effects of cDNA synthesis or preamplification (Table S6) and found a Pearson correlation of 78% (*P* = 1.36 × 10^−4^), thus validating findings.

### Predictive Modeling Through Machine Learning Analysis

To predict each patient's probability of having the disease (ie, IRBD, DLB, or PD) versus being a control, we computed binary classification analyses using the caret framework in R, specifying age, sex, and RT‐qPCR expression values of the 12 validated serum DEmiRs as predictors. Multifold imputations of missing data were performed by the predictive mean matching method implemented in the mouse R package. Numerical variables were centered and normalized, resulting in a standard distribution of mean 0 and variance 1. The gradient boosting machine (GBM) algorithm in the gbm package (v.2.1.8) was employed to classify patients in the two groups (disease vs control), using leave‐one‐out cross‐validation to cut for potential overfitting. Variable selection was applied to each training dataset using the least absolute shrinkage and selection operator (Lasso) method in the glmnet package (v.2.0‐18) (Table S7) so that only those features with a regression Beta greater than 0 were subsequently downstreamed to the GBM classifier. To address imbalanced class distributions, we performed random upsampling of the minority class on each training data fold so that the two classes acquired the same frequency using the upSample function from the caret. To evaluate the predictive performance of this model classifier, we inferred the accuracy, sensitivity, specificity, and positive and negative predictive values using the crossval package. We assessed discriminative ability by calculating the area under the curve (AUC) on a receiver operating characteristic (ROC) curve using the pROC package.

### Biological Enrichment Analysis

To investigate the biological functions and localization of the identified DEmiR, we performed a restrictive overrepresented biological enrichment analysis using only the 12 DEmiRs that were deregulated at all longitudinal time points of both DaT‐negative and DaT‐positive IRBD and also in LBD (let‐7c‐5p, miR‐19b‐3p, miR‐425‐5p, miR‐140‐3p, miR‐22‐3p, miR‐4505, miR‐221‐3p, miR‐24‐3p, miR‐25‐3p, miR‐29c‐3p, miR‐361‐5p, and miR‐451a). To this end, we used the GeneOntology option from the miEAA 2.0 software, applying gene annotations from the miRTarBase database as earlier described^23^ and the localization (RNALocate) (https://ccb-compute2.cs.uni-saarland.de/mieaa2).

## Results

We first profiled overall miRNA levels in serum samples from patients with IRBD and healthy control patients as an approach to screen global miRNA expression variation and identify specific candidate miRNAs for validation. Compared with control patients, by genome‐wide miRNA expression analysis, we found 116 candidate miRNAs in DaT‐negative IRBD patients and 50 miRNAs in DaT‐positive IRBD patients, which surpassed the fold‐change threshold greater than |1.5| and a *P* value <0.05 (Table S3, Fig. S2). Given the higher sensitivity of RT‐qPCR,^24^, ^25^ we defined DEmiRs based only on the stringent criteria of RT‐qPCR validation with a fold‐change > |1.5| and an FDR adjusted *P* < 0.05. To this end, we selected a total of 10 miRNAs among the topmost deregulated miRNAs identified at the genome‐wide profiling, including seven miRNAs from DaT‐positive IRBD patients (let‐7c‐5p, miR‐140‐3p, miR‐24‐3p, miR‐25‐3p, hsa‐miR‐361‐5p, miR‐425‐5p, and miR‐451a) and three from DaT‐negative IRBD patients (miR‐1207‐5p, miR‐1227‐5p, and miR‐3613‐5p).

Next, by RT‐qPCR, we quantified the relative expression levels of the selected miRNAs in DaT‐negative IRBD, DaT‐positive IRBD, and LBD phenoconverted patients (PD and DLB), all compared with healthy controls (Table 2). Of these, we validated seven DEmiRs in DaT‐negative and DaT‐positive IRBD patients (let‐7c‐5p, miR‐1227‐5p, miR‐24‐3p, miR‐25‐3p, miR‐361‐5p, miR‐425‐5p, and miR‐451a). All of these DEmiRs were also deregulated in LBD phenoconverted patients, except miR‐1227‐5p. Of the remaining three, miR‐140‐3p was differentially expressed in LBD and DaT‐positive IRBD patients, but not in DaT‐negative IRBD patients. In addition, miR‐1207‐5p and miR‐3613‐5p were not validated in any group. Besides candidates from the array, we also quantified the serum expression levels of five miRNA candidates earlier associated with PD alone (miR‐22‐3p, miR‐221‐3p, miR‐29c‐3p, and miR‐4505)^7^, ^21^, ^22^ or with PD and IRBD (miR‐19b‐3p).^8^, ^9^ We found that miR‐19b‐3p, miR‐22‐3p, miR‐221‐3p, and miR‐29c‐3p were DEmiRs in all DaT‐negative IRBD, DaT‐positive IRBD, and LBD patients. Of these, miR‐19b‐3p and miR‐29c‐3p were topmost deregulated in most comparisons (Table 2). In addition, miR‐4505 was a DEmiR only in LBD, but not in IRBD. Globally, by cross‐sectional analysis, we identified 13 DEmiRs showing continued deregulation across the entire RBD continuum, including DaT‐negative IRBD, DaT‐positive IRBD, and IRBD patients who phenoconverted into PD/DLB.

**TABLE 2:** RT‐qPCR assessment of microRNA expression levels in serum samples from DaT‐negative IRBD, DaT‐positive IRBD, and LBD patients (PD and DLB) as compared with healthy controls

miRNA	DaT‐negative IRBD						DaT‐positive IRBD							
	Baseline		Time point 2		Time point 3		Baseline		Time point 2		Time point 3		LBD	
	FC	Adj. *P*	FC	Adj. *P*	FC	Adj. *P*	FC	Adj. *P*	FC	Adj. *P*	FC	Adj. *P*	FC	Adj. *P*
miR‐19b‐3p	5.07	0.0002	11.59	<1.0 × 10^−6^	10.18	4.0 × 10^−6^	5.45	0.0001	12.17	<1.0 × 10^−6^	14.00	<1.0 × 10^−6^	11.44	1.0 × 10^−6^
miR‐29c‐3p	5.67	0.0002	11.24	<1.0 × 10^−6^	13.78	<1.0 × 10^−6^	5.17	0.0003	12.92	<1.0 × 10^−6^	15.15	<1.0 × 10^−6^	9.41	<1.0 × 10^−6^
miR‐451a	6.00	0.0006	17.79	0.0007	16.66	4.6 × 10^−5^	7.72	0.0003	44.54	<1.0 × 10^−6^	42.32	<1.0 × 10^−6^	18.42	<1.0 × 10^−6^
miR‐425‐5p	−4.22	0.0011	9.97	1.0 × 10^−6^	5.93	0.0015	−3.64	0.0024	12.92	<1.0 × 10^−6^	12.41	<1.0 × 10^−6^	18.36	<1.0 × 10^−6^
miR‐22‐3p	3.41	0.0014	13.56	<1.0 × 10^−6^	14.14	2.0 × 10^−6^	3.67	0.0005	14.04	<1.0 × 10^−6^	17.07	<1.0 × 10^−6^	12.86	<1.0 × 10^−6^
miR‐24‐3p	3.51	0.0017	17.99	<1.0 × 10^−6^	12.85	1.3 × 10^−5^	3.85	0.0006	23.50	<1.0 × 10^−6^	21.37	<1.0 × 10^−6^	15.14	<1.0 × 10^−6^
miR‐221‐3p	2.47	0.0020	8.30	<1.0 × 10^−6^	7.08	0.0001	2.54	0.0012	10.81	<1.0 × 10^−6^	10.86	<1.0 × 10^−6^	8.44	<1.0 × 10^−6^
miR‐1227‐5p	−1.78	0.0064	−1.04	0.8443	−1.04	0.9069	−1.58	0.0365	1.06	0.7864	−1.23	0.2916	−1.20	0.4466
miR‐25‐3p	3.18	0.0108	15.34	<1.0 × 10^−6^	10.01	0.0003	4.16	0.0024	18.21	<1.0 × 10^−6^	20.97	<1.0 × 10^−6^	11.83	<1.0 × 10^−6^
miR‐361‐5p	2.48	0.0229	12.51	<1.0 × 10^−6^	7.51	0.0003	3.32	0.0033	17.93	<1.0 × 10^−6^	15.41	<1.0 × 10^−6^	10.94	<1.0 × 10^−6^
let‐7c‐5p	1.81	0.0454	5.38	<1.0 × 10^−6^	4.82	0.0008	2.25	0.0024	6.27	<1.0 × 10^−6^	7.70	<1.0 × 10^−6^	6.28	1.0 × 10^−6^
miR‐140‐3p	1.82	0.2706	14.17	<1.0 × 10^−6^	9.02	0.0008	3.26	0.0034	17.65	<1.0 × 10^−6^	17.85	<1.0 × 10^−6^	14.56	1.0 × 10^−6^
miR‐4505	−1.15	0.6917	2.21	0.0068	2.41	0.0020	1.24	0.4253	3.16	0.0007	3.46	5.0 × 10^−6^	1.89	0.0011
miR‐1207‐5p	−1.55	0.2706	1.40	0.2978	1.36	0.3792	−1.47	0.2305	1.61	0.1666	1.15	0.6314	1.06	0.8236
miR‐3613‐5p	1.06	0.8772	1.23	0.4684	1.46	0.3140	1.16	0.5718	1.23	0.4471	1.48	0.1314	1.20	0.5155

RT‐qPCR, real‐time quantitative polymerase chain reaction; DaT, dopamine transporter single‐photon emission computed tomography imaging; IRBD, isolated rapid eye movement sleep behavior disorder; LBD, Lewy body disease (PD and DLB); PD, Parkinson's disease; DLB, dementia with Lewy bodies; FC, fold change; Adj. *P*, false discovery rate multiple‐test adjusted *P* value.

**FIG 1:**
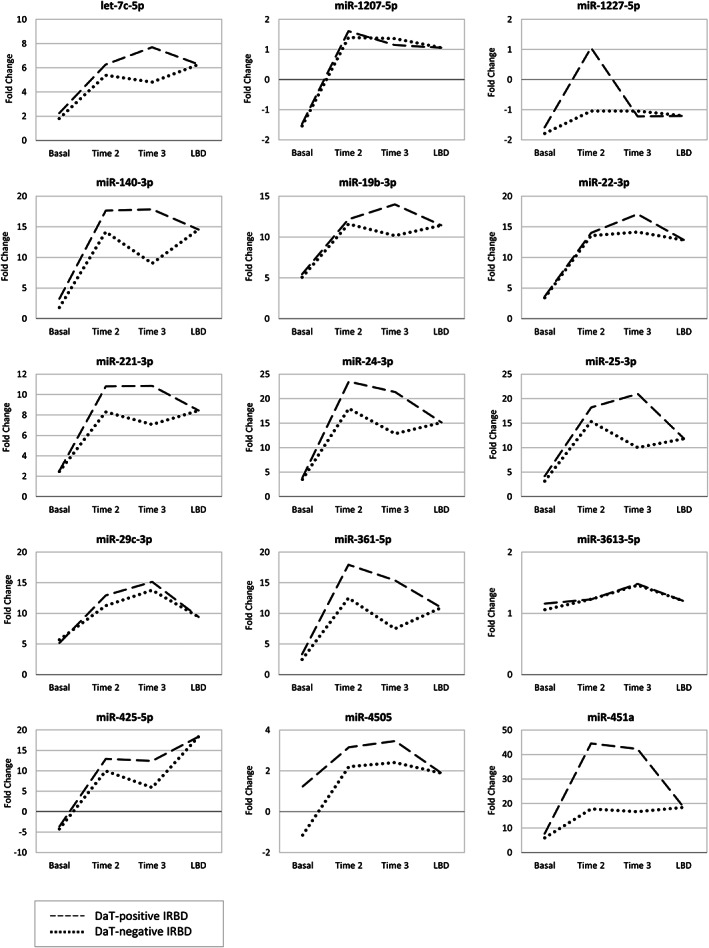
Longitudinal microRNA (miRNA) expression levels in serum from patients with dopamine transporter (DaT)‐negative idiopathic rapid eye movement sleep behavior disorder (IRBD), DaT‐positive IRBD, and LBD compared with healthy controls. Dashed line, DaT‐positive IRBD; dotted line, DaT‐negative IRBD. LBD, Lewy body disease (Parkinson's disease and dementia with Lewy bodies).

Subsequently, we performed a follow‐up miRNA expression analysis by RT‐qPCR using longitudinal serums from DaT‐negative and DaT‐positive IRBD patients. Beyond baseline, we used two additional sera covering 26 months (Table 2, Fig. S3). Compared with controls, we found consistent longitudinal miRNA deregulation of 10 DEmiRs in DaT‐negative and DaT‐positive IRBD patients (let‐7c‐5p, miR‐19b‐3p, miR‐22‐3p, miR‐221‐3p, miR‐24‐3p, miR‐25‐3p, miR‐29c‐3p, miR‐361‐5p, miR‐425‐5p, and miR‐451a). Overall, fold‐change expression values were larger at time points 2 and 3 than baseline, and expression differences were stronger in DaT‐positive than DaT‐negative IRBD (Fig. 1). We also observed that miR‐140‐3p and miR‐4505, which were not deregulated at baseline, emerged as DEmiR in the longitudinal follow‐up. Lastly, miR‐1207‐5p, miR‐3613‐5p, and miR‐1227‐5p did not show longitudinal expression differences, so we excluded these miRNAs from the enrichment analyses. Altogether, we found longitudinal miRNA expression changes of 12 DEmiRs in DaT‐negative and DaT‐positive IRBD, which had consistent deregulation in PD/DLB‐converted patients (let‐7c‐5p, miR‐19b‐3p, miR‐140, miR‐22‐3p, miR‐221‐3p, miR‐24‐3p, miR‐25‐3p, miR‐29c‐3p, miR‐361‐5p, miR‐425‐5p, miR‐4505, and miR‐451a).

**FIG 2:**
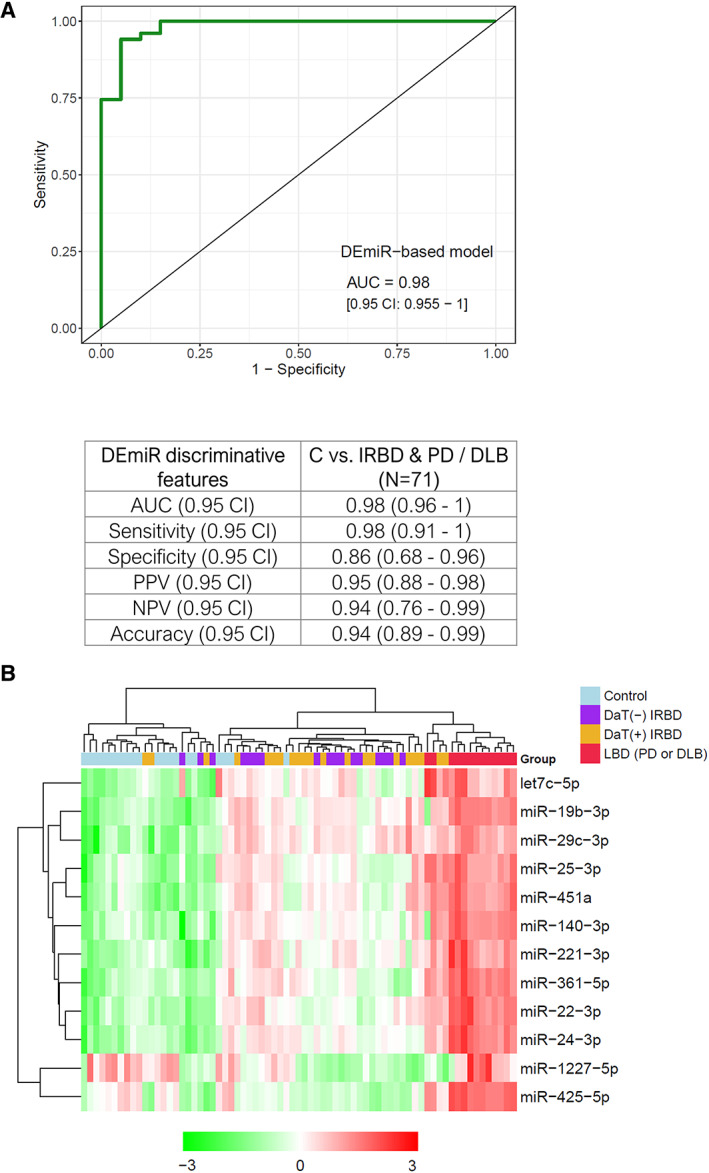
Differential expression and diagnostic accuracy of the 12 miRNA biosignatures. (**A**) Receiver operating characteristic (ROC) curve showing the discriminative ability from the 12 miRNA biosignatures to predict the subject probability of disease, IRBD, or PD/DLB versus being a healthy control subject (n = 71). Table showing performance metrics obtained through machine learning analysis for binary classification of individuals within the disease or control groups. (**B**) Unsupervised hierarchical clustering displaying real‐time quantitative polymerase chain reaction expression levels of the 12 miRNA signatures. Red pixels indicate miRNA overexpression, and green decreased expression levels. AUC, area under the curve; CI, confidence interval; DEmiR, differentially expressed microRNA; DLB, dementia with Lewy bodies; IRBD, idiopathic rapid eye movement sleep behavior disorder; LBD, Lewy body disease (PD and DLB); NPV, negative predictive value; PD, Parkinson's disease; PPV, positive predictive value. [Color figure can be viewed at wileyonlinelibrary.com]

With the goal of discerning patients with the disease (n = 51; i.e., IRBD and PD or DLB patients) from controls (n = 20) and evaluating the discriminative potential of miRNAs, we performed predictive modeling based on the serum expression levels of the 12 experimentally validated DEmiRs identified in the cross‐sectional analysis, adjusted by age and sex. During leave‐one‐out cross‐validation, predictor features selected by Lasso in 100% of the training sets were age, sex, miR‐1227‐5p, miR‐425‐5p, and miR‐451a (Table S7). The 12‐DEmiR‐based model classified 67/71 patients in the correct group (94% accuracy; 95% confidence interval [CI]: 89–99%), with a sensitivity for true disease cases of 98% (95% CI: 91–100%) and a specificity for true controls of 86% (95% CI: 68–96%). In terms of discriminative ability, ROC curve analysis showed an AUC of 0.98 (95% CI: 0.96–1) (Fig. 2A). Regarding individual miRNAs, age‐ and sex‐adjusted AUC values ranged from 0.54 up to 0.85 (Table S8), and the topmost discriminant individual miRNAs distinguishing IRBD or LBD were miR‐29c‐3p, miR‐1227‐5p, and miR‐24‐3p, with AUCs of 0.85, 0.76, and 0.76, respectively. In summary, by predictive modeling, we found a biosignature of 12 DEmiRs, which alone, without further clinical or DaT‐SPECT imaging inputs, efficiently predicted IRBD and PD or DLB clinical outcomes.

To further test the miRNA biosignature discriminative power for unbiased subject classification, we performed an unsupervised hierarchical clustering analysis using R. Consistent with the clinical status, we observed clear segregation into three main subclusters, including controls, patients with IRBD, and PD/DLB phenoconverted patients (Fig. 2B). Driven by these results, we next interrogated specific miRNA differences occurring within the IRBD continuum. We found no miRNA level differences between DaT‐negative and DaT‐positive IRBD, indicating no main relation between miRNA levels and DaT‐SPECT status. However, we observed statistically significant expression differences for all 12 biosignature miRNAs between DaT‐positive IRBD and PD/DLB patients, thus suggesting an association between the identified 12 miRNAs and the clinical phenoconversion into α‐synucleinopathy (Table S9). These findings indicate that IRBD exhibits differential serum miRNA profiles compared with controls and PD/DLB phenoconverted patients, supporting that the identified serum miRNA biosignature can be informative as a disease progression biomarker for IRBD‐initiated α‐synucleinopathies.

Lastly, we conducted a biological enrichment analysis to explore the functionality of the genes targeted by the miRNAs differentially expressed at all longitudinal time points. We found substantial deregulation at biological terms previously involved in clinically manifested PD, such as ubiquitin‐dependent protein degradation, inositol triphosphate signaling, apoptosis,^26^ myelin sheaths, synaptic transmission, postsynaptic assembly, synaptic maturation, and synaptic vesicles, among others. Regarding localization, we found exosomes, microvesicles, and extracellular vesicles among the top terms (Table 3).

**TABLE 3:** Functional enrichment of the predicted genes targeted by the 12 differentially expressed microRNAs in patients with IRBD showing the 15 top enriched terms

Enriched terms	*P* value	Adj. *P* value
Subcategory: Gene Ontology (GO) biological processes		
Positive regulation of ubiquitin‐dependent protein catabolic process	5.8 × 10^−9^	4.9 × 10^−5^
Positive regulation of TRAIL‐activated apoptotic signaling pathway	4.2 × 10^−8^	1.9 × 10^−4^
Inositol‐1,3,4,5‐tetrakisphosphate 3‐phosphatase activity	3.8 × 10^−7^	2.4 × 10^−4^
Myelin sheath adaxonal region	2.9 × 10^−7^	2.4 × 10^−4^
Negative regulation of keratinocyte migration	2.9 × 10^−7^	2.4 × 10^−4^
Negative regulation of synaptic vesicle clustering	2.9 × 10^−7^	2.4 × 10^−4^
Neuron–neuron synaptic transmission	2.0 × 10^−7^	2.4 × 10^−4^
Phosphatidylinositol‐3,4,5‐trisphosphate 3‐phosphatase activity	3.0 × 10^−7^	2.4 × 10^−4^
Phosphatidylinositol‐3,4‐bisphosphate 3‐phosphatase activity	2.9 × 10^−7^	2.4 × 10^−4^
Postsynaptic density assembly	9.9 × 10^−8^	2.4 × 10^−4^
Prepulse inhibition	4.0 × 10^−7^	2.4 × 10^−4^
Regulation of cellular component size	2.9 × 10^−7^	2.4 × 10^−4^
Rhythmic synaptic transmission	4.0 × 10^−7^	2.4 × 10^−4^
Synapse maturation	3.4 × 10^−7^	2.4 × 10^−4^
Brain morphogenesis	7.2 × 10^−7^	2.4 × 10^−4^
Subcategory: KEGG pathways		
Amyotrophic lateral sclerosis (ALS)	1.3 × 10^−4^	0.0112
Apoptosis—multiple species	1.5 × 10^−4^	0.0112
Carbohydrate digestion and absorption	2.1 × 10^−4^	0.0112
Epithelial cell signaling in *Helicobacter pylori* infection	2.5 × 10^−4^	0.0112
Fc epsilon RI signaling pathway	2.1 × 10^−4^	0.0112
GnRH secretion	1.5 × 10^−4^	0.0112
Toll‐like receptor signaling pathway	1.0 × 10^−4^	0.0112
PD‐L1 expression and PD‐1 checkpoint pathway in cancer	3.6 × 10^−4^	0.0145
Central carbon metabolism in cancer	6.7 × 10^−4^	0.0238
African trypanosomiasis	0.0010	0.0247
C‐type lectin receptor signaling pathway	9.5 × 10^−4^	0.0247
Proteasome	0.0011	0.0247
Pyrimidine metabolism	0.0010	0.0247
Salmonella infection	8.3 × 10^−4^	0.0247
TGF‐ß signaling pathway	0.0012	0.0247
TNF signaling pathway	0.0012	0.0247
Subcategory: localization (RNALocate)		
Exosome	2.0 × 10^−8^	2.0 × 10^−7^
Cytoplasm	1.3 × 10^−7^	6.5 × 10^−7^
Microvesicle	6.9 × 10^−7^	2.3 × 10^−6^
Mitochondrion	1.4 × 10^−5^	3.5 × 10^−5^
Nucleus	2.2 × 10^−5^	4.3 × 10^−5^
Extracellular vesicle	2.8 × 10^−5^	4.6 × 10^−5^
Circulating	4.8 × 10^−5^	6.9 × 10^−5^
Nucleolus	1.5 × 10^−4^	1.9 × 10^−4^
Exosome	0.0167	0.0185
Microvesicle	0.0194	0.0194

IRBD, isolated rapid eye movement sleep behavior disorder; KEGG, Kyoto Encyclopedia of Genes and Genomes; TGF‐ß, transforming growth factor ß; TNF, tumor necrosis factor; TRAIL, tumor necrosis factor‐related apoptosis‐inducing ligand; GnRH, Gonadotropin‐Releasing Hormone; PD‐L1, programmed cell death ligand 1; PD‐1, programmed cell death 1.

## Discussion

We profiled serum miRNA levels in a Spanish IRBD cohort stratified into the seriated disease progression stages of DaT‐negative IRBD, DaT‐positive IRBD with a dopaminergic deficit,^27^ and PD/DLB phenoconverted patients in which the disease had been initiated with IRBD. We uncovered a 12‐DEmiR biosignature that shows sustained and differential deregulation profiles across prodromal DaT‐negative and DaT‐positive IRBD (including their longitudinal follow‐up), and patients with manifest PD or DLB (let‐7c‐5p, miR‐1227‐5p, miR‐19b‐3p, miR‐140‐3p, miR‐22‐3p, miR‐221‐3p, miR‐24‐3p, miR‐25‐3p, miR‐29c‐3p, miR‐361‐5p, miR‐425‐5p, and miR‐451a). The identified miRNA biosignature exhibited high diagnostic accuracy for discriminating patients with IRBD and PD or DLB from controls, achieving an AUC of 98%.

To the best of our knowledge, this is the first miRNA study in patients with IRBD characterized by DaT‐SPECT with longitudinal follow‐up, but two previous studies explored miRNAs in IRBD. A first study using candidate miRNAs reported the deregulation of miR‐19b‐3p in IRBD serum (n = 104) up to 5 years before α‐synucleinopathy diagnosis.^9^ Another deep sequencing study using whole blood from a mixed cohort of IRBD and hyposmic subjects (n = 223) reported 500 miRNAs.^10^ Four of the 12 biosignature miRNAs here were topmost deregulated in the earlier reports (miR‐19b‐3p, miR‐29c‐3p, miR‐221‐5p, and miR‐140‐5p).^9^, ^10^ Complementing these studies, we found a miRNA biosignature informative for all IRBD progression stages, including IRBD and PD/DLB phenoconverted patients. Our findings align with studies showing early molecular deregulation in prodromal stages of α‐synucleinopathies. Thus, recent works in IRBD observed misfolded α‐synuclein in the cerebrospinal fluid (CSF) before developing motor and cognitive impairment.^28^ Another study in IRBD showed the presence of phosphorylated α‐synuclein aggregates in the minor labial salivary glands.^29^ Akin to these studies, our findings support that early molecular changes associated with α‐synucleinopathies occur at early IRBD prodromal stages and can antedate the clinical manifestations of PD and DLB.

In PD and DLB, developing disease prediction strategies ahead of the motor and cognitive manifestations is a challenge for the early neuroprotective intervention.^30^, ^31^ By machine learning, we evaluated the disease prediction capacity of the combined expression levels from the 12 miRNA biosignatures adjusted by age and sex to estimate each proband's probability of disease, IRBD and PD/DLB versus being control and without any further clinical or imaging input. The miRNA biosignature achieved a high discriminative capacity with an AUC of 98% (95% CI: 89–99%) and distinguished prodromal and manifest PD or DLB from controls more accurately than other previously proposed candidate biomarkers, including α‐synuclein.^8^, ^32^, ^33^, ^34^, ^35^ The predictive model also accurately classified both positive and negative individuals (94% accuracy), specifically identifying true positives (98% sensitivity). However, while the fraction of false negatives was only 2%, the false‐positive rate was 14% (86% specificity). Therefore, it is important to emphasize the need to develop more specific strategies to capture true negatives. In this sense, regarding the most clinically relevant miRNAs detected by the Lasso algorithm using a feature importance selection, only four of the six miRNAs selected in more than 90% of training folds displayed AUC scores greater than 75% at the individual level (miR‐1227‐5p, miR‐425‐5p, miR‐451a, and miR‐221‐3p).^32^, ^33^ Altogether, these findings support the proof‐of‐principle that, beyond clinical or imaging markers such as DaT‐SPECT, specific serum miRNA deregulation alone can be informative to predict the presence of IRBD and PD or DLB.

Based on our findings, an exciting interpretation is that the identified biosignature could hold potential as progression or phenoconversion biomarkers for patients within the LBD spectrum. Our miRNA findings are novel in IRBD but largely consistent with previous studies in manifest PD. Ten of the 12 biosignature miRNAs were earlier deregulated in different PD or DLB biospecimens. In LBD peripheral tissues, miR‐19b‐3p,^8^, ^36^, ^37^ miR‐29c‐3p,^7^, ^8^, ^37^, ^38^ miR‐221‐3p,^6^, ^7^, ^37^ miR‐24‐3p,^5^, ^36^ and miR‐451a^37^ were reported in PD serum; miR‐19b‐3p, miR‐29c‐3p, and miR‐361 in PD or early‐stage PD peripheral blood mononuclear cells^33^, ^39^, ^40^; miR‐451 in PD leukocytes^41^; miR‐25 in DLB platelets^42^; and miR‐22,^21^ miR‐4505,^22^ and miR‐140‐3p^10^ in PD whole blood. Other biosignature miRNAs were described in the central nervous system (CNS), such as miR‐19b‐3p, miR‐22, miR‐29c,^43^ and miR‐24^44^ in PD CSF; or miR‐221 and miR‐425‐5p^45^, ^46^ in PD brain. Lastly, let‐7c was not described in PD previously, but other members of the let‐7 family showed deregulation in PD.^32^, ^43^, ^47^, ^48^, ^49^ In summary, most of the 12 biosignature miRNAs were earlier reported in α‐synucleinopathy.^5^, ^6^, ^7^, ^10^, ^21^, ^22^, ^33^, ^36^, ^37^, ^38^, ^39^, ^40^, ^41^, ^42^ This is relevant given that, if validated, our findings may have implications for other PD at‐risk cohorts, such as individuals with hyposmia or LRRK2 or GBA asymptomatic mutation carriers.

Functionally, the target genes from the 12 longitudinal DEmiRs are involved in ubiquitin‐dependent protein degradation, inositol triphosphate signaling, apoptosis,^26^ and neural terms such as myelin sheaths, synaptic transmission, postsynaptic assembly, synaptic maturation, or synaptic vesicle. Although the function and origin of serum miRNAs are not clear,^50^ the terms found collectively support a plausible role in the CNS. Conceptually, brain‐derived exosomes crossing the brain–blood barrier represent a potential biomarker source for neurodegenerative diseases,^51^ which is in line with our finding of exosome as the top localization term. In this context, the biological enrichment analysis in our study pinpointed a plausible brain or CSF origin of at least some of the biosignature miRNAs, thus supporting the concept that minimally invasive serum miRNAs could mirror pathophysiological processes occurring at the CNS in α‐synucleinopathies. Future neuronal exosome studies in PD serum should address this question, especially if interrogating PD prodromal stages. Illustratively, a recent study reported increased α‐synuclein at neuronal exosomes from IRBD serum preceding the diagnosis of PD, persisting during disease progression, and predicting PD clinical outcomes versus atypical parkinsonisms.^52^

Despite the promising identification of a novel miRNA biosignature that is informative of different disease progression stages in α‐synucleinopathies, our study has limitations. First, we performed a restrictive miRNA expression analysis filtering in only miRNAs validated by RT‐qPCR, but additional candidates could also be nominated for validation. Second, our miRNA analyses have not been corrected for IRBD medication, such as melatonin, which has been shown to interact with specific miRNAs in mice.^53^, ^54^ Third, ours is the first serum miRNA longitudinal study in IRBD characterized by DaT‐SPECT, but the follow‐up (26 months) was limited. Fourth, the power to discriminate between subtypes of α‐synucleinopathy in our predictive modeling requires larger LBD sample sizes. Indeed, given the retrospective character of this study, further validation analyses in independent, larger cohorts of patients are warranted to ensure the predictive ability of this serum miRNA biosignature. Lastly, it has to be mentioned that the reproducibility of PD miRNA to date has been limited because of ancestry differences, lack of gold standard normalization methods, and reduced cohort sizes. In the absence of gold standard normalizers across populations,^18^ we applied both endogenous and exogenous controls^17^, ^55^ as an improved strategy that can increase cross‐laboratory miRNA reproducibility.

In summary, we identified a serum miRNA biosignature that is informative in discriminating healthy controls, patients with IRBD, and patients with PD/DLB and holds potential as a disease progression biomarker. If validated, our findings may have implications for disease prediction strategies and early detection of α‐synucleinopathies such as PD and DLB. Further studies in other prodromal PD/DLB or PD/DLB at‐risk cohorts are warranted.

## Author Roles

1. Conception and design of the study: Conception (R.F.‐S., M.E.), Organization (A.I., R.F.‐S., M.E.), Execution (M. Soto, S.L., M.F., M. Serradell, P.M.); Patient recruitment (A.I., M. Serradell, C.G., M.J.M., J.S.).
2. Acquisition and analysis of data: Design (R.F.‐S., M.E., J.C.), Execution (M. Soto, S.L., M.F., M. Serradell, P.M.), Interpretation (M. Soto, A.I., J.C., R.F.‐S., M.E.); Review and Critique (A.I., J.C., R.F.‐S., M.E., M. Serradell, C.G., M.J.M., J.S.).
3. Drafting of the manuscript or figures: Writing of the first draft (M. Soto, A.I., S.L., J.C., R.F.‐S., M.E.), Draft editing (M. Soto, A.I., S.L., J.C., R.F.‐S., M.E.), Review and Critique (all authors).

## Financial Disclosures

R.F.‐S. and M.E. received funding from The Michael J. Fox Foundation for Parkinson's Research (MJFF) (grant MJFF‐000858). R.F.‐S. was supported by the Instituto de Salud Carlos III (grant PI20/00659). M.E. received funding from the Instituto de Salud Carlos III (grant PI20/00259).

## Supporting information


**FIG. S1** Quality control assessment of serum miRNA by electropherogram.

**FIG. S2** Venn diagrams representing the number of candidate miRNAs from the genome‐wide miRNA analysis and DEmiR from the RT‐qPCR analysis in DaT‐negative IRBD, DaT‐positive IRBD and LBD compared to controls. IRBD, idiopathic rapid eye movement sleep behaviour disorder; DaT, DaT‐SPECT imaging; DaT(−), DaT‐negative IRBD patients; DaT(+), DaT‐positive IRBD patients; LBD, Lewy body disease (PD and DLB)

**FIG. S3** Individual cross‐sectional and longitudinal ∆Ct values of DaT‐negative IRBD, DaT‐positive IRBD, LBD and controls as assessed by RT‐qPCR. DaT‐negative IRBDs are represented in blue, DaT‐positive IRBD in orange, LBD in purple and controls in green.

**TABLE S1** Number of subjects treated for RBD and LBD and range of dose. IRBD, isolated rapid eye movement sleep behaviour disorder; DaT, DaT‐SPECT imaging; LBD, Lewy body disease (PD and LBD)
**TABLE S2**: Timepoint intervals between longitudinal follow‐up sampling per each subject. Number of months between baseline sampling and timepoints 2 and 3 in DaT‐negative and DaT‐positive IRBD subjects. IRBD, isolated rapid eye movement sleep behaviour disorder; DaT, DaT‐SPECT imaging
**TABLE S3**: Candidate differentially expressed miRNAs identified by genome‐wide microRNA expression analysis. (A) DaT‐negative IRBD versus controls. (B) DaT‐positive IRBD versus controls. IRBD, isolated rapid eye movement sleep behaviour disorder; DaT, DaT‐SPECT imaging; FC, Fold change
**TABLE S4**: Details of commercially available TaqMan Fast Advanced miRNA assays for assessment of miRNA expression levels by real‐time quantitative PCR (RT‐qPCR). (A) Assays used for RT‐qPCR assessment of miRNA levels. (B) Assays discarded due to poor RT‐qPCR amplification
**TABLE S5**: RT‐qPCR assessment of 10 selected miRNAs identified by genome‐wide miRNA expression analysis. IRBD, isolated rapid eye movement sleep behaviour disorder; RT‐qPCR, real‐time quantitative PCR; FC, fold change. (*) Differentially expressed miRNAs (DEmiR) from the array validated by RT‐qPCR with the same fold change direction and a statistically significant *P*‐value
**TABLE S6**: Technical validation of the real‐time quantitative PCR (RT‐qPCR) longitudinal analyses. Replication of the longitudinal RT‐qPCR assessment of miRNA expression levels in serum samples from DaT‐positive and DaT‐negative IRBD patients as compared to controls. IRBD, isolated rapid eye movement sleep behaviour disorder; DaT, DaT‐SPECT imaging; FC, fold change; adj. *P*, false discovery rate (FDR) multiple‐testing adjusted *P*‐values
**TABLE S7**: Frequency of selection by LASSO of each miRNA included in machine‐learning analysis folds (N = 71).
**TABLE S8**: Performance metrics achieved by machine‐learning analysis for detection of IRBD, PD and DLB (N = 71). (*) AUC values are adjusted by age and sex in the machine‐learning classifier. (**) *P*‐values obtained by Mann–Whitney *U* test. AUC, area under the curve; CI, confidence interval; Adj. *P*‐value, FDR multiple testing adjusted *P*‐value; IRBD, isolated rapid eye movement sleep behaviour disorder
**TABLE S9**: RT‐qPCR assessment of miRNA expression levels in serum samples within the IRBD continuum. IRBD, isolated rapid eye movement sleep behaviour disorder; LBD, Lewy body disease (PD and DLB); DaT, DaT‐SPECT imaging; DaT(−) IRBD, DaT‐negative IRBD patients; DaT(+) IRBD, DaT‐positive IRBD patients; FC, fold change; Adj. *P*, FDR multiple‐test adjusted *P*‐value

## Data Availability

Data available on request from the authors
