# Supplementary material for: Serum MicroRNAs Predict Isolated Rapid Eye Movement Sleep Behavior Disorder and Lewy Body Diseases
Source: Mov Disord. 2022 Aug 12;37(10):2086–98. doi: 10.1002/mds.29171 (PMC9804841; doi:10.1002/mds.29171)
Supplement: Supplementary file 1 — FIG. S1 Quality control assessment of serum miRNA by electropherogram. [file MDS-37-2086-s002.pdf]

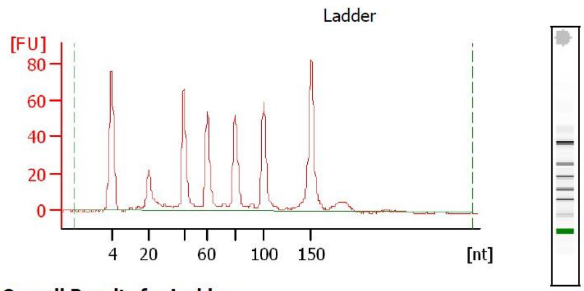

Overall Results for Ladder

Result Flagging Color:

Result Flagging Label: Number of Peaks: 6

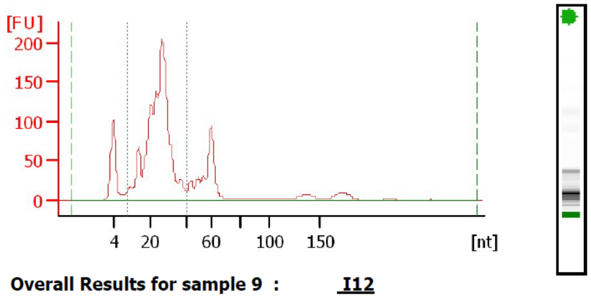

Overall Results for sample 9 : I12

Small RNA Concentration [pg/µl]:	10.005,7
miRNA Concentration [pg/µl]:	8.940,2
miRNA / Small RNA Ratio [%]:	89
Result Flagging Color:	
Result Flagging Label:	89 % miRNA; Concentration: 8940.20 pg/µl

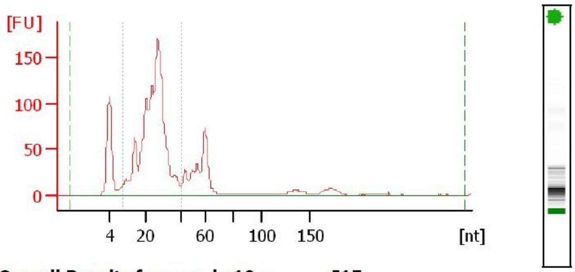

Overall Results for sample 10 : I15

Small RNA Concentration [pg/µl]:	8.243,6
miRNA Concentration [pg/µl]:	7.363,3
miRNA / Small RNA Ratio [%]:	89
Result Flagging Color:	
Result Flagging Label:	89 % miRNA; Concentration: 7363.30 pg/µl

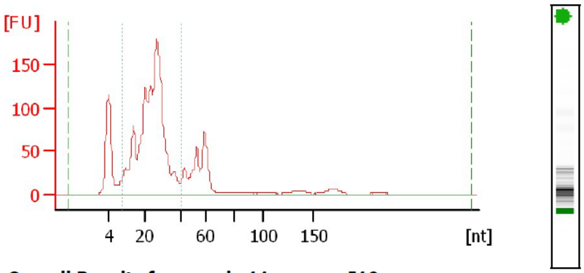

Overall Results for sample 11 : I19

Small RNA Concentration [pg/µl]:	7.796,2
miRNA Concentration [pg/µl]:	6.937,4
miRNA / Small RNA Ratio [%]:	89
Result Flagging Color:	
Result Flagging Label:	89 % miRNA; Concentration: 6937.40 pg/µl

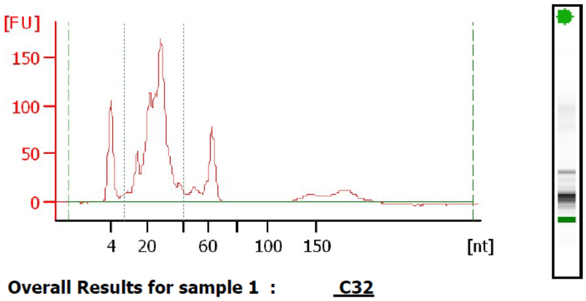

Overall Results for sample 1 : C32

Small RNA Concentration [pg/µl]:	7.862,2
miRNA Concentration [pg/µl]:	7.093,7
miRNA / Small RNA Ratio [%]:	90
Result Flagging Color:	
Result Flagging Label:	90 % miRNA; Concentration: 7093.70 pg/µl

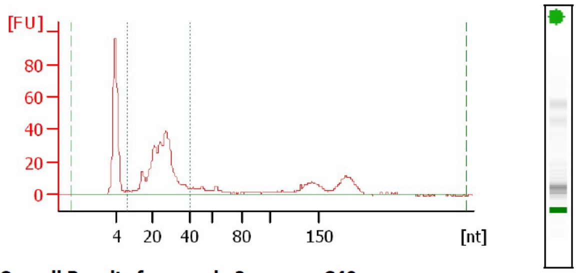

Overall Results for sample 2 : C40

Small RNA Concentration [pg/µl]:	2.369,4
miRNA Concentration [pg/µl]:	2.103,6
miRNA / Small RNA Ratio [%]:	89
Result Flagging Color:	
Result Flagging Label:	89 % miRNA; Concentration: 2103.60 pg/µl

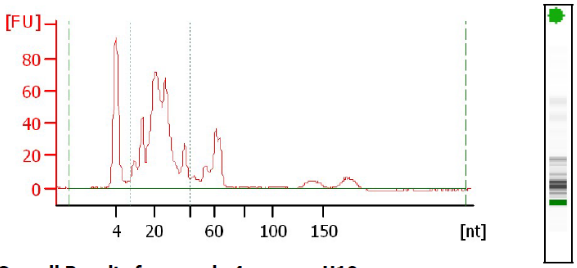

Overall Results for sample 4 : H10

Small RNA Concentration [pg/µl]:	4.684,8
miRNA Concentration [pg/µl]:	4.222,5
miRNA / Small RNA Ratio [%]:	90
Result Flagging Color:	
Result Flagging Label:	90 % miRNA; Concentration: 4222.50 pg/µl

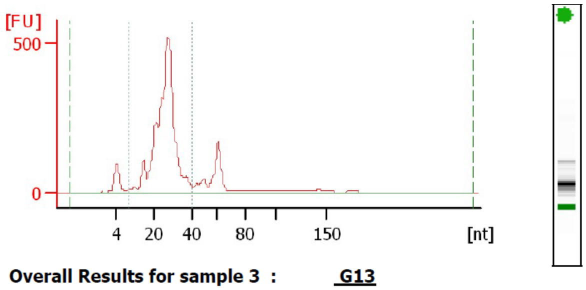

Overall Results for sample 3 : G13

Small RNA Concentration [pg/µl]:	22.296,2
miRNA Concentration [pg/µl]:	20.736,4
miRNA / Small RNA Ratio [%]:	93
Result Flagging Color:	
Result Flagging Label:	93 % miRNA; Concentration: 20736.40 pg/µl

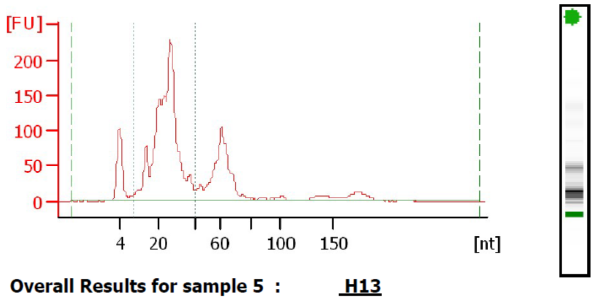

Overall Results for sample 5 : **H13**

Small RNA Concentration [pg/µl]: 11.061,4
miRNA Concentration [pg/µl]: 9.566,3
miRNA / Small RNA Ratio [%]: 86
Result Flagging Color:
Result Flagging Label: 86 % miRNA; Concentration: 9566.30 pg/µl

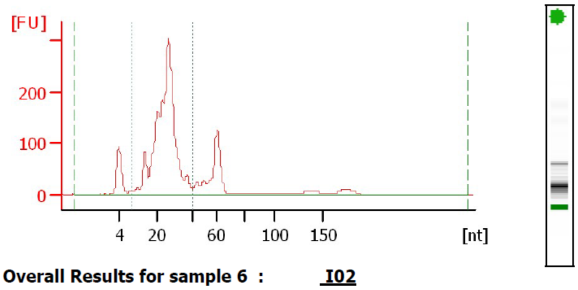

Overall Results for sample 6 : **I02**

Small RNA Concentration [pg/µl]: 14.327,6
miRNA Concentration [pg/µl]: 13.102,9
miRNA / Small RNA Ratio [%]: 91
Result Flagging Color:
Result Flagging Label: 91 % miRNA; Concentration: 13102.90 pg/µl

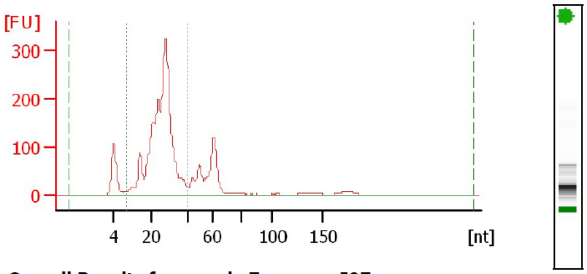

Overall Results for sample 7 : **I07**

Small RNA Concentration [pg/µl]: 13.185,8
miRNA Concentration [pg/µl]: 11.847,7
miRNA / Small RNA Ratio [%]: 90
Result Flagging Color:
Result Flagging Label: 90 % miRNA; Concentration: 11847.70 pg/µl

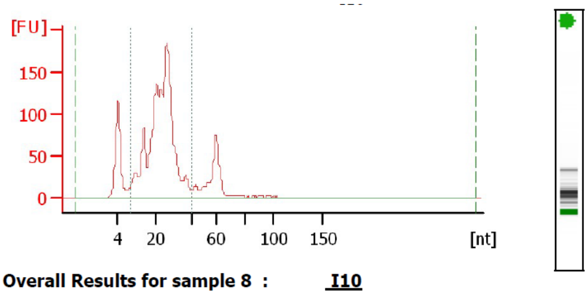

Overall Results for sample 8 : **I10**

Small RNA Concentration [pg/µl]: 7.945,7
miRNA Concentration [pg/µl]: 7.398,0
miRNA / Small RNA Ratio [%]: 93
Result Flagging Color:
Result Flagging Label: 93 % miRNA; Concentration: 7398 pg/µl

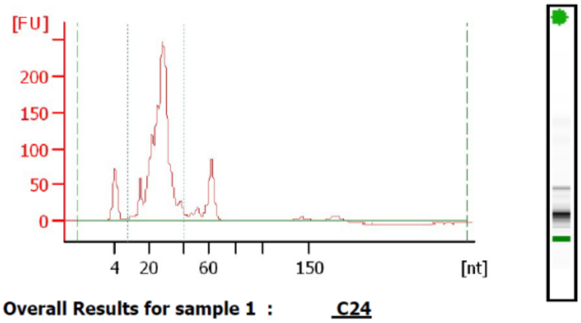

Overall Results for sample 1 : **C24**

Small RNA Concentration [pg/µl]: 12.374,7
miRNA Concentration [pg/µl]: 11.537,6
miRNA / Small RNA Ratio [%]: 93
Result Flagging Color:
Result Flagging Label: 93 % miRNA; Concentration: 11537.60 pg/µl

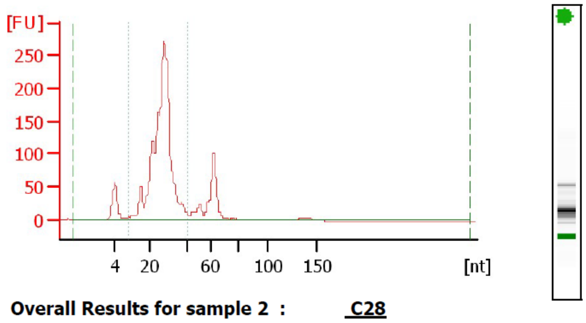

Overall Results for sample 2 : **C28**

Small RNA Concentration [pg/µl]: 16.768,4
miRNA Concentration [pg/µl]: 15.626,8
miRNA / Small RNA Ratio [%]: 93
Result Flagging Color:
Result Flagging Label: 93 % miRNA; Concentration: 15626.80 pg/µl

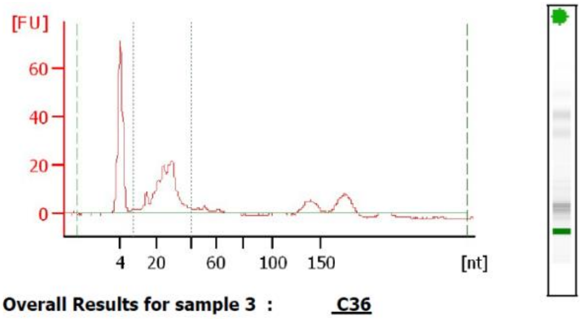

Overall Results for sample 3 : **C36**

Small RNA Concentration [pg/µl]: 1.553,2
miRNA Concentration [pg/µl]: 1.364,5
miRNA / Small RNA Ratio [%]: 88
Result Flagging Color:
Result Flagging Label: 88 % miRNA; Concentration: 1364.50 pg/µl

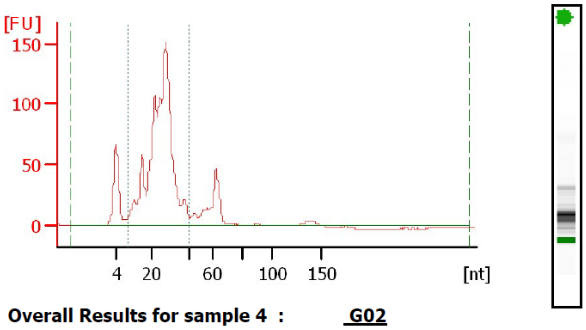

Overall Results for sample 4 : **G02**

Small RNA Concentration [pg/µl]: 9.559,0
miRNA Concentration [pg/µl]: 8.997,4
miRNA / Small RNA Ratio [%]: 94
Result Flagging Color:
Result Flagging Label: 94 % miRNA; Concentration: 8997.40 pg/µl

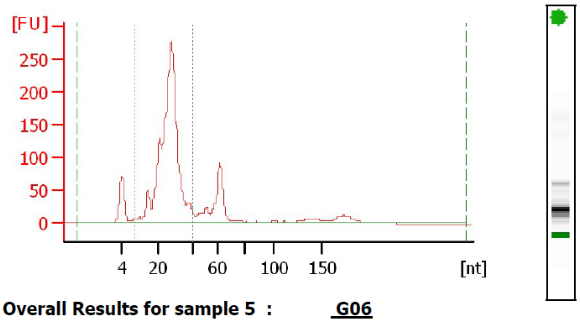

Overall Results for sample 5 : G06

Small RNA Concentration [pg/µl]: 13.888,0
miRNA Concentration [pg/µl]: 12.710,1
miRNA / Small RNA Ratio [%]: 92
Result Flagging Color:
Result Flagging Label: 92 % miRNA; Concentration: 12710.10 pg/µl

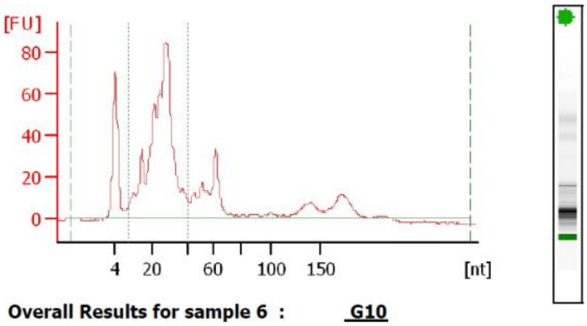

Overall Results for sample 6 : G10

Small RNA Concentration [pg/µl]: 5.815,4
miRNA Concentration [pg/µl]: 4.957,9
miRNA / Small RNA Ratio [%]: 85
Result Flagging Color:
Result Flagging Label: 85 % miRNA; Concentration: 4957.90 pg/µl

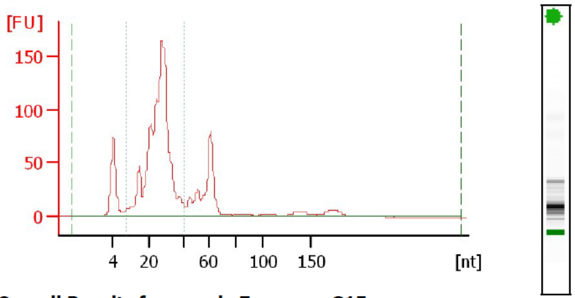

Overall Results for sample 7 : G15

Small RNA Concentration [pg/µl]: 9.109,0
miRNA Concentration [pg/µl]: 8.144,7
miRNA / Small RNA Ratio [%]: 89
Result Flagging Color:
Result Flagging Label: 89 % miRNA; Concentration: 8144.70 pg/µl

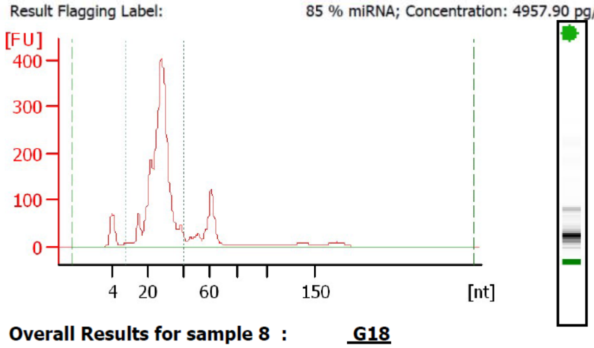

Overall Results for sample 8 : G18

Small RNA Concentration [pg/µl]: 20.041,1
miRNA Concentration [pg/µl]: 18.467,0
miRNA / Small RNA Ratio [%]: 92
Result Flagging Color:
Result Flagging Label: 92 % miRNA; Concentration: 18467 pg/µl

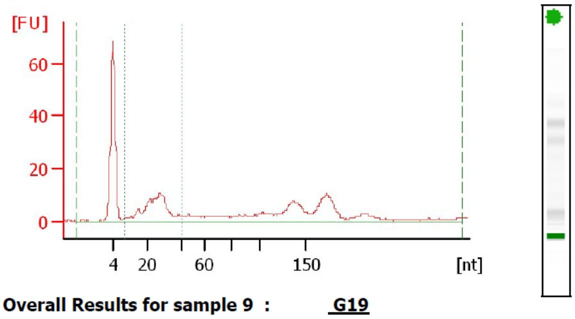

Overall Results for sample 9 : G19

Small RNA Concentration [pg/µl]: 1.271,3
miRNA Concentration [pg/µl]: 791,4
miRNA / Small RNA Ratio [%]: 62
Result Flagging Color:
Result Flagging Label: 62 % miRNA; Concentration: 791.40 pg/µl

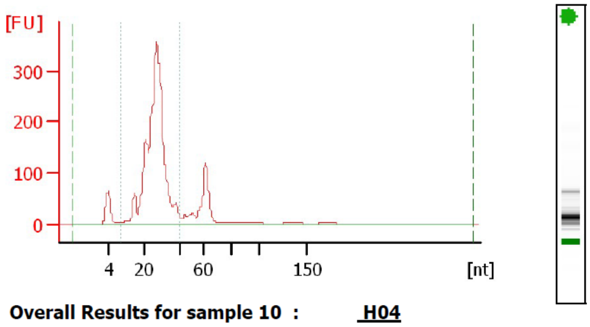

Overall Results for sample 10 : H04

Small RNA Concentration [pg/µl]: 19.632,5
miRNA Concentration [pg/µl]: 18.040,6
miRNA / Small RNA Ratio [%]: 92
Result Flagging Color:
Result Flagging Label: 92 % miRNA; Concentration: 18040.60 pg/µl

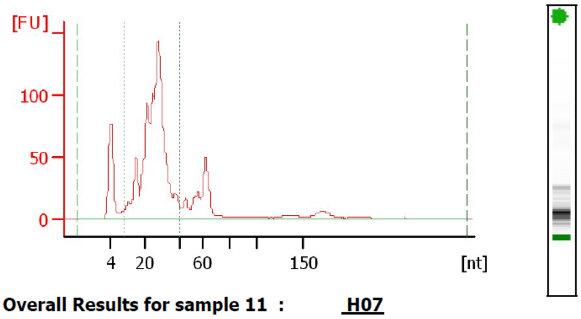

Overall Results for sample 11 : H07

Small RNA Concentration [pg/µl]: 7.798,3
miRNA Concentration [pg/µl]: 6.998,0
miRNA / Small RNA Ratio [%]: 90
Result Flagging Color:
Result Flagging Label: 90 % miRNA; Concentration: 6998 pg/µl

Suppl. Fig. 1
