# Supplementary material for: Serum MicroRNAs Predict Isolated Rapid Eye Movement Sleep Behavior Disorder and Lewy Body Diseases
Source: Mov Disord. 2022 Aug 12;37(10):2086–98. doi: 10.1002/mds.29171 (PMC9804841; doi:10.1002/mds.29171)
Supplement: Supplementary file 2 — FIG. S2 Venn diagrams representing the number of candidate miRNAs from the genome‐wide miRNA analysis and DEmiR from the RT‐qPCR analysis in DaT‐negative IRBD, DaT‐positive IRBD and LBD compared to controls. IRBD, idiopathic rapid eye movement sleep behaviour disorder; DaT, DaT‐SPECT imaging; DaT(−), DaT‐negative IRBD patients; DaT(+), DaT‐positive IRBD patients; LBD, Lewy body disease (PD and DLB) [file MDS-37-2086-s001.pdf]

Genome-wide candidate miRNA

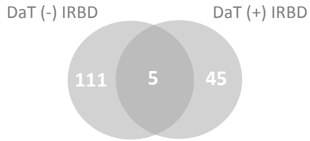

Groups	Total	miRNAs
DaT (+) IRBD vs. C<br>DaT (-) IRBD vs. C	5	miR-8084 miR-6780b-5p miR-6741-5p miR-3201 miR-4701-3p
DaT (-) IRBD vs. C	111	miR-4707-5p miR-6126 miR-6848-5p miR-6816-5p miR-7108-5p miR-4758-5p miR-6716-5p miR-4486 miR-4539 miR-4689 miR-1275 miR-4710 miR-6763-5p miR-6729-5p miR-4793-3p miR-92b-5p miR-4505 miR-6771-5p miR-1228-5p miR-6786-5p miR-3960 miR-6779-5p miR-6752-5p miR-5093 miR-3197 mir-6089-1 miR-1343-5p miR-185-3p miR-1909-3p miR-595 mir-6089-2 miR-4498 miR-6775-5p miR-4466 miR-2861 miR-6727-5p miR-7110-5p miR-4492 miR-4507 miR-1587 miR-4695-5p miR-6891-5p miR-762 miR-3648 miR-1469 miR-149-3p miR-6756-5p miR-6821-5p miR-3621 miR-6769b-5p miR-3613-5p miR-328-5p miR-4632-5p miR-6858-5p miR-1268b mir-4679-1 miR-3656 miR-663a miR-6850-5p miR-7107-5p miR-4281 miR-6749-5p miR-8072 miR-3620-5p miR-6722-3p miR-6869-5p miR-4745-5p miR-3178 miR-1207-5p miR-6798-5p miR-1237-5p miR-6805-5p mir-1913 miR-4270 miR-6799-5p miR-4739 miR-1227-5p miR-7150 miR-4459 miR-6791-5p miR-5001-5p miR-4440 miR-3135b miR-4463 miR-6789-5p miR-4690-5p miR-937-5p miR-4484 mir-4466 miR-4649-5p miR-4651 miR-320d miR-4433b-3p miR-6824-5p miR-1225-5p miR-4429 mir-4281 miR-1908-5p miR-3141 miR-6743-5p miR-4508 miR-6860 miR-4462 miR-4763-3p miR-6787-5p miR-3940-5p miR-1268a miR-1233-5p miR-4433-3p miR-6724-5p miR-4734
DaT (+) IRBD vs. C	45	miR-151a-5p miR-361-5p miR-23a-3p miR-4454 miR-185-5p miR-150-5p miR-4530 miR-744-5p miR-22-3p miR-122-5p miR-17-5p miR-24-3p miR-4467 mir-550a-3 miR-26a-5p miR-342-3p miR-20a-5p miR-652-3p miR-25-3p let-7b-5p mir-550a-1 miR-6732-5p miR-23b-3p miR-193a-5p miR-877-5p miR-3128 let-7d-5p miR-297 let-7c-5p miR-4487 miR-191-5p miR-451a mir-7515 miR-638 miR-140-3p miR-106a-5p miR-7114-5p miR-16-5p miR-126-3p miR-425-5p miR-5787 miR-103a-3p miR-93-5p miR-107 mir-550a-2

Cross-sectional RT-qPCR DEmiR

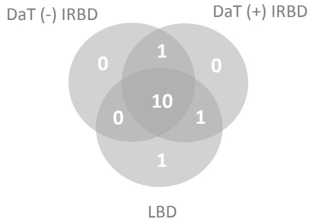

Groups	Total	miRNAs
DaT (+) IRBD vs. C<br>DaT (-) IRBD vs. C<br>LBD vs. C	10	miR-19b-3p miR-425-5p miR-24-3p miR-29c-3p miR-221-3p miR-25-3p miR-361-5p miR-451a let-7c-5p miR-22-3p
DaT (+) IRBD vs. C<br>DaT (-) IRBD vs. C	1	miR-1227-5p
DaT (+) IRBD vs. C<br>LBD vs. C	1	miR-140-3p
LBD vs. C	1	miR-4505

Longitudinal RT-qPCR DEmiR DAT (-) IRBD vs. C

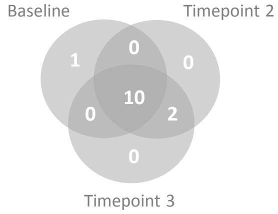

Groups	Total	miRNAs
Baseline<br>Timepoint 1<br>Timepoint 2	10	miR-19b-3p miR-425-5p miR-24-3p miR-29c-3p miR-221-3p miR-25-3p miR-361-5p miR-451a let-7c-5p miR-22-3p
Timepoint 2<br>Timepoint 3	2	miR-140-3p miR-4505
Baseline	1	miR-1227-5p

Longitudinal RT-qPCR DEmiR DAT (+) IRBD vs. C

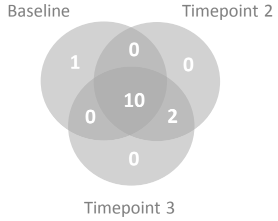

Groups	Total	miRNAs
Baseline<br>Timepoint 1<br>Timepoint 2	11	miR-19b-3p miR-425-5p miR-24-3p miR-29c-3p miR-221-3p miR-140-3p miR-25-3p miR-361-5p miR-451a let-7c-5p miR-22-3p
Timepoint 1<br>Timepoint 3	1	miR-4505
Baseline	1	miR-1227-5p

Suppl. Fig. 2
