# Supplementary figures and images for: Serum MicroRNAs Predict Isolated Rapid Eye Movement Sleep Behavior Disorder and Lewy Body Diseases
Source: Mov Disord. 2022 Aug 12;37(10):2086–98. doi: 10.1002/mds.29171 (PMC9804841; doi:10.1002/mds.29171)

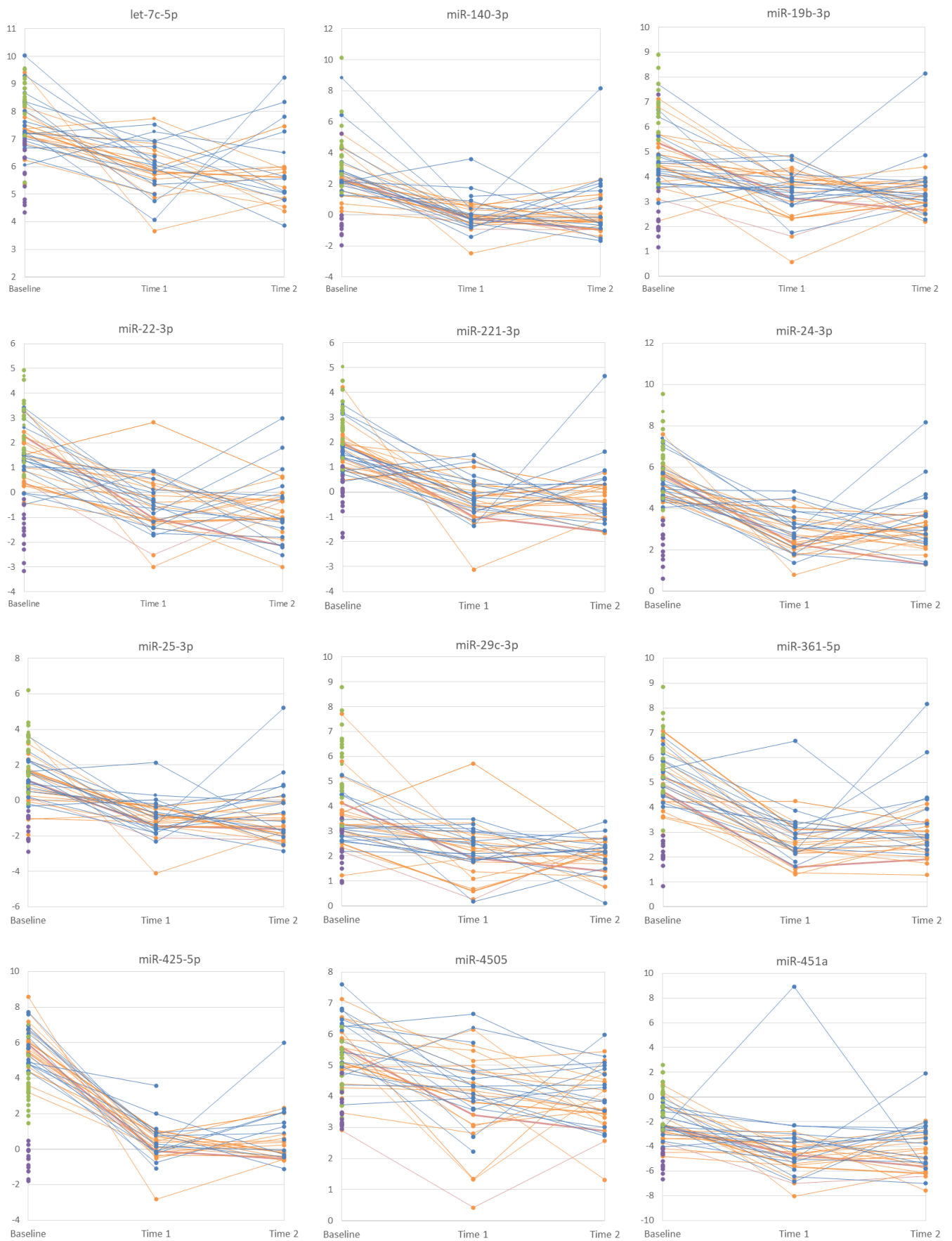

Suppl. Fig. 3

Supplement: Supplementary file 3 — FIG. S3 Individual cross‐sectional and longitudinal ∆Ct values of DaT‐negative IRBD, DaT‐positive IRBD, LBD and controls as assessed by RT‐qPCR. DaT‐negative IRBDs are represented in blue, DaT‐positive IRBD in orange, LBD in purple and controls in green. [file MDS-37-2086-s004.pdf]
