# Supplementary material for: Serum MicroRNAs Predict Isolated Rapid Eye Movement Sleep Behavior Disorder and Lewy Body Diseases
Source: Mov Disord. 2022 Aug 12;37(10):2086–98. doi: 10.1002/mds.29171 (PMC9804841; doi:10.1002/mds.29171)
Supplement: Supplementary file 4 — TABLE S1 Number of subjects treated for RBD and LBD and range of dose. IRBD, isolated rapid eye movement sleep behaviour disorder; DaT, DaT‐SPECT imaging; LBD, Lewy body disease (PD and LBD) TABLE S2: Timepoint intervals between longitudinal follow‐up sampling per each subject. Number of months between baseline sampling and timepoints 2 and 3 in DaT‐negative and DaT‐positive IRBD subjects. IRBD, isolated rapid eye movement sleep behaviour disorder; DaT, DaT‐SPECT imaging TABLE S3: Candidate differentially expressed miRNAs identified by genome‐wide microRNA expression analysis. (A) DaT‐negative IRBD versus controls. (B) DaT‐positive IRBD versus controls. IRBD, isolated rapid eye movement sleep behaviour disorder; DaT, DaT‐SPECT imaging; FC, Fold change TABLE S4: Details of commercially available TaqMan Fast Advanced miRNA assays for assessment of miRNA expression levels by real‐time quantitative PCR (RT‐qPCR). (A) Assays used for RT‐qPCR assessment of miRNA levels. (B) Assays discarded due to poor RT‐qPCR amplification TABLE S5: RT‐qPCR assessment of 10 selected miRNAs identified by genome‐wide miRNA expression analysis. IRBD, isolated rapid eye movement sleep behaviour disorder; RT‐qPCR, real‐time quantitative PCR; FC, fold change. (*) Differentially expressed miRNAs (DEmiR) from the array validated by RT‐qPCR with the same fold change direction and a statistically significant P‐value TABLE S6: Technical validation of the real‐time quantitative PCR (RT‐qPCR) longitudinal analyses. Replication of the longitudinal RT‐qPCR assessment of miRNA expression levels in serum samples from DaT‐positive and DaT‐negative IRBD patients as compared to controls. IRBD, isolated rapid eye movement sleep behaviour disorder; DaT, DaT‐SPECT imaging; FC, fold change; adj. P, false discovery rate (FDR) multiple‐testing adjusted P‐values TABLE S7: Frequency of selection by LASSO of each miRNA included in machine‐learning analysis folds (N = 71). TABLE S8: Performance metrics achieved by ma [file MDS-37-2086-s003.docx]

**SUPPLEMENTARY TABLE 1: Number of subjects treated for RBD and LBD and range of dose.**

|  | **RBD treatment** | | | | **LBD treatment** | | | | | |
| --- | --- | --- | --- | --- | --- | --- | --- | --- | --- | --- |
|  | **Clonazepam** | | **Melatonin** | | **L-DOPA** | | **Cholinesterase inhibitors** | | | |
|  |  |  |  |  |  |  | **Rivastigmine** | | **Donepezil** | |
|  | **Treated Nr. (%)** | **Dose (mg)** | **Treated Nr. (%)** | **Dose (mg)** | **Treated Nr. (%)** | **LEDD** | **Treated Nr. (%)** | **Dose (mg)** | **Treated Nr. (%)** | **Dose (mg)** |
| DaT(-) IRBD baseline (n=17) | 12 (70.6) | 0.25-2 | 1 (5.9) | 4 | 0 | - | 0 | - | 0 | - |
| Timepoint 2 (n=16) | 15 (93.8) | 0.25-3 | 1 (6.3) | 2 | 0 | - | 0 | - | 0 | - |
| Timepoint 3 (n=14) | 13 (92.9) | 0.25-3 | 0 (0.0) | - | 0 | - | 0 | - | 0 | - |
| DaT(+) IRBD baseline (n=21) | 16 (76.2) | 0.25-2 | 3 (14.3) | 2-6 | 0 | - | 0 | - | 0 | - |
| Timepoint 2 (n=19) | 12 (63.2) | 0.25-2 | 7 (36.8) | 2-6 | 1 (5.3) | 100 | 0 | - | 0 | - |
| Timepoint 3 (n=19) | 11 (57.9) | 0.25-2 | 7 (36.8) | 5-6 | 1 (5.3) | 450 | 0 | - | 0 | - |
| LBD (PD and DLB) (n=13) | 9 (69.2) | 0.5-2.5 | 2 (15.4) | 2-9 | 5 (38.5) | 300-450 | 2 (15.4) | 4.6-13 | 1 (7.7) | 10 |

IRBD = isolated rapid eye movement sleep behaviour disorder; DaT = DaT-SPECT imaging; LBD = Lewy body disease (PD and LBD)

**SUPPLEMENTARY TABLE 2: Timepoint intervals between longitudinal follow-up sampling per each subject.** Number of months between baseline sampling and timepoints 2 and 3 in DaT-negative and DaT-positive IRBD subjects. IRBD = isolated rapid eye movement sleep behaviour disorder; DaT= DaT-SPECT imaging

|  | DaT-negative IRBD | | | | | | | | | | | | | | | | | | | | |
| --- | --- | --- | --- | --- | --- | --- | --- | --- | --- | --- | --- | --- | --- | --- | --- | --- | --- | --- | --- | --- | --- |
| Subject | H01 | H02 | H03 | H04 | H05 | H06 | H07 | H08 | H09 | H10 | H11 | H12 | H13 | H14 | H15 | H16 | H17 |  |  |  |  |
| Timepoint 2 | 9 | 10 | 9 | 13 | 13 | 6 | 9 | 9 | 14 | 8 | 14 | 9 | 10 | 5 | 12 | - | 12 |  |  |  |  |
| Timepoint 3 | 15 | 16 | 16 | 24 | 26 | - | 20 | 15 | 25 | 21 | 19 | 13 | 29 | 17 | 18 | - | - |  |  |  |  |
|  | **DaT-positive IRBD** | | | | | | | | | | | | | | | | | | | | |
| Subject | G01 | G02 | G03 | G04 | G05 | G06 | G07 | G08 | G09 | G10 | G11 | G12 | G13 | G14 | G15 | G16 | G17 | G18 | G19 | G20 | G21 |
| Timepoint 2 | 13 | 5 | 14 | 11 | 11 | 11 | 10 | 10 | 11 | 10 | 12 | 12 | 10 | 10 | 14 | - | 14 | 10 | 9 | 9 | - |
| Timepoint 3 | 25 | 11 | 26 | 17 | 20 | 20 | 20 | 18 | 18 | 23 | 18 | 17 | 22 | 19 | 26 | - | 26 | 21 | 15 | 18 | - |

**SUPPLEMENTARY TABLE 3: Candidate differentially expressed miRNAs identified by genome-wide microRNA expression analysis.** (**A**) DaT-negative IRBD vs. controls. (**B**) DaT-positive IRBD vs. controls. IRBD = isolated rapid eye movement sleep behaviour disorder; DaT = DaT-SPECT imaging; FC = Fold change

**A**

| **DaT-negative IRBD vs. controls** | | | | | |
| --- | --- | --- | --- | --- | --- |
| **miRNA** | **P-value** | **FC** | **miRNA** | **P-value** | **FC** |
| miR-1207-5p | 0.0004 | -3.4591 | miR-6724-5p | 0.0187 | -5.2830 |
| miR-3613-5p | 0.0004 | -4.8195 | miR-1228-5p | 0.0188 | -6.5628 |
| miR-6749-5p | 0.0013 | -3.8441 | mir-4281 | 0.0191 | -2.5978 |
| miR-1225-5p | 0.0015 | -2.6884 | mir-4466 | 0.0191 | -2.3140 |
| miR-3135b | 0.0020 | -3.3210 | miR-4492 | 0.0191 | -3.7386 |
| miR-1268a | 0.0020 | -4.0370 | miR-4508 | 0.0195 | -4.3001 |
| miR-4433-3p | 0.0022 | -2.8057 | miR-5093 | 0.0198 | -3.6453 |
| miR-7107-5p | 0.0030 | -3.4487 | miR-6860 | 0.0207 | -1.6281 |
| miR-4651 | 0.0031 | -4.7370 | miR-6798-5p | 0.0228 | -3.8951 |
| miR-4632-5p | 0.0043 | -3.4045 | miR-6850-5p | 0.0230 | -3.3948 |
| miR-1275 | 0.0045 | -2.6125 | miR-6743-5p | 0.0231 | -3.8223 |
| miR-6780b-5p | 0.0049 | -2.8125 | miR-762 | 0.0235 | -5.9579 |
| miR-6787-5p | 0.0053 | -3.3145 | miR-4710 | 0.0241 | -1.9819 |
| miR-6741-5p | 0.0055 | -2.7941 | miR-1908-5p | 0.0249 | -3.8674 |
| miR-6848-5p | 0.0057 | -2.2715 | miR-4701-3p | 0.0254 | -3.3770 |
| miR-4486 | 0.0059 | -3.6694 | mir-1913 | 0.0255 | -2.1930 |
| miR-1227-5p | 0.0061 | -4.5322 | miR-4281 | 0.0257 | -5.6829 |
| miR-6775-5p | 0.0062 | -2.9418 | mir-4679-1 | 0.0257 | 2.1690 |
| miR-6126 | 0.0064 | -11.6036 | miR-8072 | 0.0266 | -3.8344 |
| miR-1268b | 0.0065 | -3.3554 | miR-4462 | 0.0268 | -2.3962 |
| miR-937-5p | 0.0067 | -2.2224 | miR-4429 | 0.0270 | -2.6096 |
| miR-7150 | 0.0067 | -2.1668 | miR-3197 | 0.0279 | -2.9470 |
| miR-4440 | 0.0071 | -10.4155 | miR-1469 | 0.0285 | -2.4667 |
| miR-4498 | 0.0074 | -2.1742 | miR-7108-5p | 0.0292 | -5.5311 |
| miR-3141 | 0.0077 | -3.7493 | miR-663a | 0.0297 | -4.3441 |
| miR-6756-5p | 0.0081 | -3.0460 | miR-92b-5p | 0.0301 | -2.0062 |
| miR-7110-5p | 0.0083 | -10.3706 | miR-4539 | 0.0315 | -1.6012 |
| miR-4690-5p | 0.0083 | -1.7339 | miR-6799-5p | 0.0316 | -2.1169 |
| miR-4689 | 0.0088 | -2.3884 | miR-6805-5p | 0.0328 | -4.7530 |
| miR-4463 | 0.0090 | -6.2556 | miR-149-3p | 0.0330 | -5.5593 |
| miR-6716-5p | 0.0092 | -2.5374 | miR-3656 | 0.0333 | -4.8674 |
| miR-4433b-3p | 0.0092 | -4.4368 | miR-3960 | 0.0334 | -3.2700 |
| miR-4649-5p | 0.0095 | -3.5703 | miR-6779-5p | 0.0340 | -2.0326 |
| miR-3648 | 0.0097 | -1.9334 | mir-6089-1 | 0.0342 | -5.0746 |
| miR-6824-5p | 0.0104 | -2.8603 | mir-6089-2 | 0.0342 | -5.0746 |
| miR-328-5p | 0.0105 | -5.2601 | miR-6891-5p | 0.0344 | -1.8823 |
| miR-4505 | 0.0105 | -2.7027 | miR-6786-5p | 0.0360 | -4.4784 |
| miR-6752-5p | 0.0110 | -5.8512 | miR-3201 | 0.0361 | 5.7072 |
| miR-4758-5p | 0.0111 | -3.2209 | miR-8084 | 0.0362 | 5.3057 |
| miR-185-3p | 0.0116 | -5.0231 | miR-2861 | 0.0362 | -3.6982 |
| miR-3178 | 0.0117 | -5.8236 | miR-5001-5p | 0.0370 | -3.4264 |
| miR-3620-5p | 0.0118 | -3.6626 | miR-6769b-5p | 0.0380 | -1.7772 |
| miR-6763-5p | 0.0123 | -1.7206 | miR-3621 | 0.0383 | -3.6583 |
| miR-4507 | 0.0123 | -3.5668 | miR-4793-3p | 0.0407 | -4.7293 |
| miR-6821-5p | 0.0125 | -5.3855 | miR-6729-5p | 0.0408 | -2.9794 |
| miR-6771-5p | 0.0125 | -3.3738 | miR-320d | 0.0416 | -4.6420 |
| miR-1909-3p | 0.0127 | -2.9737 | miR-4466 | 0.0431 | -3.7586 |
| miR-1587 | 0.0129 | -2.5024 | miR-6869-5p | 0.0434 | -4.0146 |
| miR-4695-5p | 0.0131 | -2.3696 | miR-1237-5p | 0.0437 | -5.0657 |
| miR-6791-5p | 0.0137 | -3.9123 | miR-4707-5p | 0.0456 | -2.7577 |
| miR-4739 | 0.0139 | -2.2166 | miR-4745-5p | 0.0459 | -4.3048 |
| miR-6722-3p | 0.0145 | -4.4306 | miR-4459 | 0.0464 | -2.1769 |
| miR-4270 | 0.0147 | -3.9392 | miR-4734 | 0.0465 | -3.9511 |
| miR-6858-5p | 0.0158 | -3.9603 | miR-4763-3p | 0.0467 | -2.7399 |
| miR-1343-5p | 0.0167 | -3.6256 | miR-595 | 0.0472 | -2.6410 |
| miR-6816-5p | 0.0172 | -7.3172 | miR-6727-5p | 0.0477 | -3.6857 |
| miR-3940-5p | 0.0177 | -6.5240 | miR-4484 | 0.0491 | -5.9006 |
| miR-1233-5p | 0.0186 | -2.8683 | miR-6789-5p | 0.0497 | -4.0448 |

**B**

| **DaT-positive IRBD vs. controls** | | | | | |
| --- | --- | --- | --- | --- | --- |
| **miRNA** | **P-value** | **FC** | **miRNA** | **P-value** | **FC** |
| miR-4530 | 3.23E-07 | 6.9268 | miR-151a-5p | 0.0037 | 3.3509 |
| miR-122-5p | 9.20E-06 | 17.1805 | miR-126-3p | 0.0042 | 2.5816 |
| miR-425-5p | 2.61E-05 | 10.2558 | miR-22-3p | 0.0043 | 3.6848 |
| miR-361-5p | 4.53E-05 | 3.1425 | miR-3201 | 0.0053 | -4.3733 |
| miR-25-3p | 4.97E-05 | 4.2740 | miR-106a-5p | 0.0064 | 3.5565 |
| let-7c-5p | 0.0001 | 3.8664 | miR-342-3p | 0.0064 | 3.1194 |
| miR-193a-5p | 0.0001 | 3.4711 | miR-20a-5p | 0.0066 | 2.9721 |
| miR-140-3p | 0.0003 | 6.4165 | miR-5787 | 0.0100 | -1.6947 |
| miR-4487 | 0.0003 | 2.1046 | let-7b-5p | 0.0111 | 4.9079 |
| miR-6732-5p | 0.0008 | -1.6071 | miR-107 | 0.0125 | 3.3149 |
| miR-23a-3p | 0.0008 | 7.5698 | mir-7515 | 0.0187 | -3.0543 |
| miR-191-5p | 0.0009 | 7.9700 | miR-7114-5p | 0.0190 | -2.0832 |
| miR-24-3p | 0.0009 | 6.7009 | miR-451a | 0.0201 | 2.8320 |
| miR-8084 | 0.0011 | -5.4270 | miR-6780b-5p | 0.0204 | -2.0387 |
| miR-26a-5p | 0.0012 | 5.0407 | miR-297 | 0.0220 | -3.7245 |
| miR-652-3p | 0.0014 | 3.1623 | miR-4454 | 0.0230 | 1.6804 |
| miR-17-5p | 0.0014 | 4.1517 | miR-16-5p | 0.0248 | 4.6549 |
| miR-23b-3p | 0.0017 | 3.2719 | miR-6741-5p | 0.0297 | -1.9861 |
| miR-185-5p | 0.0020 | 5.2206 | miR-4701-3p | 0.0316 | -2.5938 |
| miR-93-5p | 0.0022 | 5.3113 | miR-877-5p | 0.0402 | 1.5216 |
| miR-4467 | 0.0024 | 3.0629 | miR-638 | 0.0407 | -1.4231 |
| let-7d-5p | 0.0032 | 2.3475 | mir-550a-1 | 0.0431 | -1.3283 |
| miR-103a-3p | 0.0032 | 4.8721 | mir-550a-2 | 0.0431 | -1.3283 |
| miR-150-5p | 0.0036 | 2.8061 | mir-550a-3 | 0.0431 | -1.3283 |
| miR-744-5p | 0.0037 | 2.5224 | miR-3128 | 0.0447 | -2.5953 |

**SUPPLEMENTARY TABLE 4: Details of commercially available TaqMan Fast Advanced miRNA assays for assessment of miRNA expression levels by real-time quantitative PCR (RT-qPCR).** **(A)** Assays used for RT-qPCR assessment of miRNA levels. **(B)** Assays discarded due to poor RT-qPCR amplification

**A**

| **miRNA** | **Assay ID** | **Catalog number** | **miRbase Accession Number** | **Type** |
| --- | --- | --- | --- | --- |
| hsa-miR-6727-5p | 480235_mir | A25576 | MI0022572 | Endogenous Control |
| hsa-miR-320a-3p | 478594_mir | A25576 | MI0000542 | Endogenous Control |
| cel-miR-39-3p | 478293_mir | A25576 | MI0000010 | Exogenous Control |
| hsa-let-7c-5p | 478577_mir | A25576 | MI0000064 | Target |
| hsa-miR-1207-5p | 477873_mir | A25576 | MI0006340 | Target |
| hsa-miR-1227-5p | 480789_mir | A25576 | MI0006316 | Target |
| hsa-miR-140-3p | 477908_mir | A25576 | MI0000456 | Target |
| hsa-miR-19b-3p | 478264_mir | A25576 | MI0000074 | Target |
| hsa-miR-22-3p | 477985_mir | A25576 | MI0000078 | Target |
| hsa-miR-221-3p | 477981_mir | A25576 | MI0000298 | Target |
| hsa-miR-24-3p | 477992_mir | A25576 | MI0000080 | Target |
| hsa-miR-25-3p | 477994_mir | A25576 | MI0000082 | Target |
| hsa-miR-29c-3p | 479229_mir | A25576 | MI0000735 | Target |
| hsa-miR-361-5p | 478056_mir | A25576 | MI0000760 | Target |
| hsa-miR-3613-5p | 479424_mir | A25576 | MI0016003 | Target |
| hsa-miR-425-5p | 478094_mir | A25576 | MI0001448 | Target |
| hsa-miR-4505 | 477842_mir | A25576 | MI0016868 | Target |
| hsa-miR-451a | 478107_mir | A25576 | MI0001729 | Target |

**B**

| **miRNA** | **Assay ID** | **Catalog number** | **miRbase Accession Number** | **Type** |
| --- | --- | --- | --- | --- |
| has-miR-185-3p | 474832_mir | A25576 | MI0000482 | Did not amplify |
| hsa-miR-3619-5p | 479689_mir | A25576 | MI0016009 | Did not amplify |
| has-miR-4530 | 478918_mir | A25576 | MI0016897 | Did not amplify |
| has-miR-4632-5p | 479865_mir | A25576 | MI0017259 | Did not amplify |
| has-miR-6126 | 480186_mir | A25576 | MI0021260 | Did not amplify |
| hsa-miR-6741-3p | 480259_mir | A25576 | MI0022586 | Did not amplify |
| has-miR-6749-5p | 480272_mir | A25576 | MI0022594 | Did not amplify |

**SUPPLEMENTARY TABLE 5: RT-qPCR assessment of 10 selected miRNAs identified by genome-wide miRNA expression analysis.** IRBD = isolated rapid eye movement sleep behaviour disorder; RT-qPCR = real-time quantitative PCR; FC = fold change. (*) Differentially expressed miRNAs (DEmiR) from the array validated by RT-qPCR with the same fold change direction and a statistically significant P-value

|  | Microarray | | RT-qPCR | |
| --- | --- | --- | --- | --- |
|  | **FC** | **P-value** | **FC** | **Adj. P-value** |
| DaT-negative IRBD vs. controls | | | | |
| miR-1207-5p | -3.46 | 0.0004 | -1.55 | 0.2706 |
| miR-1227-5p | -4.53 | 0.0061* | -1.78 | 0.0064* |
| miR-3613-5p | -4.82 | 0.0004 | 1.06 | 0.8772 |
| DaT-positive IRBD vs. controls | | | | |
| Let-7c-5p | 3.87 | 0.0001* | 2.25 | 0.0024* |
| miR-140-3p | 6.42 | 0.0003* | 3.26 | 0.0034* |
| miR-24-3p | 6.70 | 0.0009* | 3.85 | 0.0006* |
| miR-25-3p | 4.27 | 4.97E-05* | 4.16 | 0.0024* |
| miR-361-5p | 3.14 | 4.53E-05* | 3.32 | 0.0033* |
| miR-425-5p | 10.25 | 2.61E-05 | -3.64 | 0.0024 |
| miR-451a | 2.83 | 0.0201* | 7.72 | 0.0003* |

**SUPPLEMENTARY TABLE 6: Technical validation of the real-time quantitative PCR (RT-qPCR) longitudinal analyses.** Replication of the longitudinal RT-qPCR assessment of miRNA expression levels in serum samples from DaT-positive and DaT-negative IRBD patients as compared to controls. IRBD = isolated rapid eye movement sleep behaviour disorder; DaT = DaT-SPECT imaging; FC = fold change; adj. P = false discovery rate (FDR) multiple-testing adjusted P-values

|  | DaT-negative IRBD | | | | | | DaT-positive IRBD | | | | | |
| --- | --- | --- | --- | --- | --- | --- | --- | --- | --- | --- | --- | --- |
|  | **Baseline** | | **Timepoint 2** | | **Timepoint 3** | | **Baseline** | | **Timepoint 2** | | **Timepoint 3** | |
|  | **FC** | **Adj. P** | **FC** | **Adj. P** | **FC** | **Adj. P** | **FC** | **Adj. P** | **FC** | **Adj. P** | **FC** | **Adj. P** |
| Initial analysis |  | | | | | |  | | | | | |
| miR-140-3p | 1.82 | 0.2706 | 14.17 | <1.0x10^-6^ | 7.51 | 0.0008 | 3.26 | 0.0034 | 17.65 | <1.0x10^-6^ | 17.85 | <1.0x10^-6^ |
| miR-19b-3p | 5.07 | 0.0002 | 11.59 | <1.0x10^-6^ | 4.82 | 4.0x10^-6^ | 5.45 | 0.0001 | 12.17 | <1.0x10^-6^ | 14.00 | <1.0x10^-6^ |
| miR-29c-3p | 5.67 | 0.0002 | 11.24 | <1.0x10^-6^ | 1.36 | <1.0x10^-6^ | 5.17 | 0.0003 | 12.92 | <1.0x10^-6^ | 15.15 | <1.0x10^-6^ |
| Replication |  | | | | | |  | | | | | |
| miR-140-3p | 1.77 | 0.0631 | 6.10 | 5.5x10^-5^ | 5.60 | 5.7x10^-5^ | 3.09 | 0.0014 | 14.19 | <1.0x10^-6^ | 12.68 | <1.0x10^-6^ |
| miR-19b-3p | 4.06 | 0.0005 | 10.19 | <1.0x10^-6^ | 9.44 | 2.0x10^-6^ | 5.75 | 3.2x10^-5^ | 14.32 | <1.0x10^-6^ | 17.19 | <1.0x10^-6^ |
| miR-29c-3p | 3.91 | 0.0014 | 9.09 | 2.0x10^-6^ | 7.92 | 1.3x10^-5^ | 4.65 | 0.0006 | 13.84 | <1.0x10^-6^ | 13.38 | <1.0x10^-6^ |

**SUPPLEMENTARY TABLE 7: Frequency of selection by LASSO of each miRNA included in machine-learning analysis folds (N=71).**

| **Variable** | **Nr. of folds** | **Frequency of folds (%)** |
| --- | --- | --- |
| Age at sampling | 71 | 100 |
| Gender | 71 | 100 |
| miR-1227-5p | 71 | 100 |
| miR-425-5p | 71 | 100 |
| miR-451a | 71 | 100 |
| miR-22-3p | 70 | 98.59 |
| miR-221-3p | 70 | 98.59 |
| miR-361-5p | 67 | 94.37 |
| miR-24-3p | 42 | 59.15 |
| let-7c-5p | 11 | 15.49 |
| miR-25-3p | 8 | 11.27 |
| miR-29c-3p | 6 | 8.45 |
| miR-19b-3p | 1 | 1.41 |
| miR-140-3p | 0 | 0 |

**SUPPLEMENTARY TABLE 8: Performance metrics achieved by machine-learning analysis for detection of IRBD, PD and DLB (N=71).** (*) AUC values are adjusted by age and sex in the machine-learning classifier. (**) P-values obtained by Mann-Whitney U test. AUC = area under the curve; CI = confidence interval; Adj. P-value = FDR multiple testing adjusted P-value; IRBD = isolated rapid eye movement sleep behaviour disorder

|  | **AUC* (0.95 CI)** | **Fold-change** | **P-value**** | **Adj. P-value** | **Regulation** |
| --- | --- | --- | --- | --- | --- |
| let-7c-5p | 0.68 (0.52 - 0.85) | 2.63 | <0.0001 | <0.0001 | Upregulated |
| miR-1227-5p | 0.76 (0.60 - 0.91) | -1.84 | 0.0001 | <0.0001 | Downregulated |
| miR-140-3p | 0.66 (0.48 - 0.83) | 3.64 | <0.0001 | <0.0001 | Upregulated |
| miR-19b-3p | 0.74 (0.60 - 0.89) | 5.20 | <0.0001 | <0.0001 | Upregulated |
| miR-22-3p | 0.58 (0.43 - 0.73) | 3.51 | <0.0001 | 0.0001 | Upregulated |
| miR-221-3p | 0.72 (0.57 - 0.87) | 3.42 | <0.0001 | <0.0001 | Upregulated |
| miR-24-3p | 0.76 (0.62 - 0.89) | 4.34 | <0.0001 | <0.0001 | Upregulated |
| miR-25-3p | 0.66 (0.51 - 0.81) | 4.04 | <0.0001 | <0.0001 | Upregulated |
| miR-29c-3p | 0.85 (0.77 - 0.94) | 5.55 | <0.0001 | <0.0001 | Upregulated |
| miR-361-5p | 0.54 (0.40 - 0.69) | 3.34 | <0.0001 | <0.0001 | Upregulated |
| miR-425-5p | 0.75 (0.58 - 0.91) | -1.40 | 0.056 | 0.3496 | Downregulated |
| miR-451a | 0.75 (0.63-0.88) | 6.68 | <0.0001 | <0.0001 | Upregulated |

**SUPPLEMENTARY TABLE 9: RT-qPCR assessment of miRNA expression levels in serum samples within the IRBD continuum.** IRBD = isolated rapid eye movement sleep behaviour disorder; LBD = Lewy body disease (PD and DLB); DaT = DaT-SPECT imaging; DaT(-) IRBD = DaT-negative IRBD patients; DaT(+) IRBD = DaT-positive IRBD patients; FC = fold change; Adj. P = FDR multiple-test adjusted P-value

|  | | DaT(+) IRBD vs. DaT(-) IRBD | | LBD vs. DaT(+) IRBD | |
| --- | --- | --- | --- | --- | --- |
|  | **FC** | **Adj. P** | **FC** | **Adj. P** |  |
| let7c-5p | | 1.16 | 0.8624 | 3.00 | 0.0039 |
| miR1227-5p | | 1.23 | 0.4361 | 2.67 | 0.0101 |
| miR140-3p | | 1.56 | 0.4361 | 5.80 | 0.0006 |
| miR19b-3p | | 1.11 | 0.8624 | 4.22 | 0.0002 |
| miR22-3p | | 1.04 | 0.8624 | 7.36 | 0.0009 |
| miR221-3p | | 1.04 | 0.8624 | 3.61 | 0.0037 |
| miR24-3p | | 1.07 | 0.8624 | 7.87 | 0.0011 |
| miR25-3p | | 1.62 | 0.4361 | 4.13 | 0.0006 |
| miR29c-3p | | 1.09 | 0.8624 | 2.09 | 0.0067 |
| miR361-5p | | 1.48 | 0.4361 | 5.63 | 0.0001 |
| miR451a | | 1.69 | 0.4361 | 4.52 | 0.0016 |
